# Distinctive Patterns of Gut *Bifidobacterium* Diversity in Mongolian Adults: Regional Variation and Dairy Intake Associations

**DOI:** 10.3390/nu18172816

**Published:** 2026-08-28

**Authors:** Tuul Nyambal, Khulan Lkhamsuren, Alexander Hübner, Ser-Od Khuygaa, Sabri Bromage, Raphaela Stahl, Matthäus Rest, Björn Reichhardt, Ariunzul Battulga, Sodbileg Odonchimed, Davaalkham Dambadarjaa, Khosbayar Tulgaa, Christina Warinner, Soninkhishig Tsolmon

**Affiliations:** 1 Department of Nutrition, School of Public Health, Mongolian National University of Medical Science, Ulaanbaatar 14210, Mongolia; tuul.n@mnums.edu.mn; 2 School of Public Health, Mongolian National University of Medical Science, Ulaanbaatar 14210, Mongolia; lettertolana@gmail.com (K.L.); serod@mnums.edu.mn (S.-O.K.); davaalkham@mnums.edu.mn (D.D.); 3 Department of Archaeogenetics, Max Planck Institute for Evolutionary Anthropology, 04103 Leipzig, Germany; alexander_huebner@eva.mpg.de (A.H.); raphaela_stahl@eva.mpg.de (R.S.); matthaeus.rest@unifr.ch (M.R.); christina_warinner@eva.mpg.de (C.W.); 4 Institute of Nutrition, Mahidol University, Nakhon Pathom 73170, Thailand; sabri.bro@mahidol.ac.th; 5 Department of Nutrition, Harvard T.H. Chan School of Public Health, Boston, MA 02115, USA; 6 Central Asia Seminar, Institute for Asian and African Studies, Humboldt-Universität zu Berlin, 10115 Berlin, Germany; bjoern.reichhardt@hu-berlin.de; 7 Mongolian Dietetic Association, Ulaanbaatar 16090, Mongolia; ariunzul.nutrition@gmail.com (A.B.);; 8 Department of Clinical Laboratory, School of Medicine, Mongolian National University of Medical Science, Ulaanbaatar 14210, Mongolia; khosbayar.t@mnums.edu.mn; 9 Department of Anthropology, Harvard University, Cambridge, MA 02138, USA; 10 School of Management, Mongolian University of Science and Technology, Ulaanbaatar 14191, Mongolia

**Keywords:** *Bifidobacterium* diversity, Mongolia, regional variation, alpha and beta diversity, dairy consumption

## Abstract

**Background/Objectives**: Despite increasing interest in the human gut microbiome, particularly in *Bifidobacterium*, studies focusing on traditionally living populations with high habitual dairy consumption remain limited. This study investigated species-level diversity, abundance, and regional variation of *Bifidobacterium* among healthy Mongolian adults and contextualized these findings within a global comparative framework. **Methods**: A total of 100 healthy adults were recruited from four Mongolian regions. Fecal samples were analyzed using species-resolved shotgun metagenomics, and dietary intake was assessed through a standardized food frequency questionnaire. Alpha- and beta-diversity metrics, differential abundance tests, and diet–microbe correlations were performed on members of the *Bifidobacterium* species. To assess the broader significance of the Mongolian *Bifidobacterium* profile, Shannon diversity was further compared against a curated global dataset comprising 1286 healthy adults from 29 countries using harmonized bioinformatic and statistical pipelines. **Results**: Shannon diversity within the *Bifidobacterium* genus was significantly higher in nomadic Mongolians, particularly those from Khuvsgul and Dundgobi, and strong geographic structuring was observed in beta-diversity analyses (PERMANOVA, *p* < 0.001). Nomadic populations showed higher relative abundances of *B. adolescentis* and *B. angulatum*, whereas *B. pseudocatenulatum* predominated in Ulaanbaatar. Although overall *Bifidobacterium* abundance was lowest in Bulgan, *B. longum* remained the dominant species in this region. Spearman correlation analysis with false discovery rate correction identified significant associations between dairy intake and specific *Bifidobacterium* species. Homemade yogurt and traditional dairy intake were positively associated with *B. angulatum*. *B. catenulatum* was positively associated with homemade yogurt, traditional dairy, and total fermented dairy intake, whereas factory milk intake was negatively associated with *B. angulatum* but positively associated with *B. pseudocatenulatum*. Mongolia ranked among the top eight of 30 countries for *Bifidobacterium* Shannon diversity though total *Bifidobacterium* abundance showed substantial inter-individual and regional variation. **Conclusions:** Mongolian adults exhibit relatively high *Bifidobacterium* diversity at both national and global scales. Traditional dairy consumption was associated with species-specific variation in *Bifidobacterium* composition, supporting the contribution of dietary practices to regional gut microbiota patterns.

## 1. Introduction

The stability and diversity of the gut microbiome play a crucial role in various aspects of human health [1]. The microbiome composition characterizes the gastrointestinal environment where different bacterial species stimulate or suppress the growth of others through substances secreted during their activities, exerting multiple effects on the host [2]. Among the constituents of the human microbiome, *Bifidobacterium* is of particular interest because it colonizes the gut shortly after birth, undergoes characteristic developmental shifts across the lifespan, and serves as a key biological indicator of intestinal health [3]. *Bifidobacteria* account for 3–17% of the human gut microbiome [2,4]. Common species include *B. adolescentis*, *B. angulatum*, *B. longum*, *B. bifidum*, *B. dentium*, *B. breve*, *B. animalis*, *B. catenulatum*, *B. pseudocatenulatum*, and *B. pseudolongum* [2]. Research has demonstrated that *Bifidobacterium* plays an essential role in generating short-chain fatty acids (SCFAs) and lactic acid via distinct pathways compared to *Lactobacillus*. Sufficient amounts of SCFAs lower the pH of the intestinal environment, inhibit pathogenic bacteria growth, help modulate immunity, provide colon cells with energy to proliferate and maintain the intestinal barrier, enhance intestinal motility, and regulate inflammatory responses [1,2,5].

It is well recognized that diet is one of the main environmental factors that have a pronounced effect on the microbiome, altering both composition and metabolic output [6,7]. Since *Bifidobacteria* are obligate anaerobes, they thrive in the oxygen-deprived environment of the colon, where dietary composition modulates their abundance and activity [8]. The consumption of complex carbohydrates, including nondigestible fibers and oligosaccharides, is considered a major nutritional factor in promoting *Bifidobacterium* growth and maintenance [9,10]. In contrast, so-called Western-type diets high in fat and protein are shown to hinder the development of the bifidobacterial population [9,11]. These diets often favor the growth of bile-tolerant and proteolytic bacteria, shifting the microbial balance and potentially decreasing beneficial metabolites, such as SCFA [7]. Recent dietary interventions and clinical studies have highlighted how fiber-rich foods can restore or enhance the bifidobacterial population [12,13]. Collectively, these findings indicate that dietary composition is an important determinant of *Bifidobacterium* abundance and community structure.

Alongside of the genus *Bifidobacterium*, members of Lactic acid bacteria (LAB) are recognized as important beneficial microorganisms associated with fermented dairy products and the human gastrointestinal tract, where they contribute to microbial balance and host metabolic regulation [14]. LAB are the most extensively studied genera because of their important role in food fermentation and various biological processes. Fermentation has been used for thousands of years as a strategy to transform raw materials into stable and edible food products, while its microbiology has been extensively investigated for more than a century [15]. In addition to their microbial content, dairy products may also contribute to the modulation of bifidobacterial populations. Fermented dairy foods, including yogurt, kefir, and traditional fermented milk products, contain bioactive compounds and microbial metabolites that can promote the growth and metabolic activity of beneficial gut bacteria, including *Bifidobacterium* [2,15]. Milk-derived oligosaccharides, peptides, and lactose have been reported to serve as selective substrates for certain *Bifidobacterium* species, particularly *B. longum* and *B. breve*, thereby supporting their persistence in the adult gut ecosystem [16]. Several dietary intervention studies have demonstrated that increased intake of fermented foods, including dairy products, can enhance gut microbial diversity and improve microbial stability, although these effects may vary depending on dairy type, fermentation process, and host dietary background [16,17,18].

Mongolia is one of the few countries that still sustains a significant nomadic population that follows a traditional pastoralist dietary pattern. It is well documented that animal husbandry-based subsistence living lasted for more than 5000 years without major changes until today. Dairy is one of the main food groups consumed by pastoral herders [19]. Mongolia’s ecological zones, Khangai (mountainous), Central Steppe, and Gobi Desert, exhibit distinct dietary patterns in terms of consuming different animal milks rooted in local geography and traditional nomadic practices. In the northern Khangai region (notably Khuvsgul prefecture), people predominantly consume high quantities of meat and dairy from free-ranging livestock, especially cattle and yaks. In Bulgan, the southern part of the Khangai region, fermented mare’s milk (*airag*) is a prominent part of the local diet. In contrast, the Gobi region (e.g., Dundgobi, Umnugobi) is characterized by arid desert-steppe environments where communities practice camel herding. Camel milk and its derivatives play a central role in household nutrition [20,21,22,23].

In recent years, Mongolia has undergone a transition from a traditionally nomadic lifestyle to a more sedentary one, particularly in urban areas. This shift has led to notable changes in dietary habits and lifestyle, resulting in marked differences in health-related factors, including gut microbiota composition and diversity [24,25,26].

Regional dietary habits and environmental exposures may significantly shape the composition and diversity of the *Bifidobacteria* species [3,25]. Understanding the factors influencing the *Bifidobacterium* dynamics on the species level is essential, as even subtle shifts in its composition can significantly impact human health. To fill these identified gaps and provide critical information on *Bifidobacterium* diversity in the Mongolian adult population across different geographical zones with distinctive dietary patterns, a comparative evaluation of *Bifidobacterium* species was performed using next-generation sequencing. Gut microbiota community analysis, with specific attention to the *Bifidobacterium* population, was conducted using microbiome data from subjects aged 18–84 years. The results of this study provide specific information on the diversity and variation of *Bifidobacteria* among nomadic and urban populations in Mongolia, in relation to dietary differences, particularly fermented dairy products from different animal milking.

## 2. Materials and Methods

### 2.1. Participant Recruitment and Fecal Sample Collection

One hundred participants were recruited from four regions of Mongolia: Ulaanbaatar (*n* = 50), Bulgan, Khuvsgul, and Dundgobi (*n* = 50). The urban group consisted of permanent residents of Ulaanbaatar whose families had lived in apartment housing for at least two generations. The nomadic group consisted of participants recruited from nomadic herder households in Bulgan, Khuvsgul, and Dundgobi provinces, representing the forest-steppe, mountain taiga, and steppe ecological zones, respectively and were engaged in traditional livestock husbandry, consuming predominantly self-produced animal products. In Mongolia, nomadic pastoralists predominantly reside in rural areas; however, the terms “nomadic” and “rural” are not synonymous, as not all rural residents maintain a fully nomadic lifestyle. Therefore, in this study “rural” refers to pure nomadic lifestyle. The inclusion criteria were as follows: 18 years or older; female participants were not pregnant or lactating; no history of severe diarrhea, constipation, or gastrointestinal disorders in the preceding month; no major illnesses, cognitive impairments, or mental disorders; and absence of antibiotic and probiotic use within the last three months. The study was approved by the Medical Ethics Review Committee of the Ministry of Health, Mongolia (Project Number: NO.2020/155) and the Research Ethics Review Committee of the Mongolian National University of Medical Sciences (Project Number: NO.2022/3-03). Informed consent was obtained from all participants before the commencement of the study.

Fecal samples were self-collected using sterile stool collection kits following standardized instructions. Samples were transported to the laboratory under cold-chain conditions and stored at −80 °C. For long-term stabilization and shipment, a portion of each sample was applied onto Whatman FTA^®^ cards (Cytiva, Marlborough, MA, USA), which lyse cells on contact and preserve nucleic acids at ambient temperature. After drying, cards were sealed with desiccant and transported in accordance with biospecimen handling guidelines [27,28].

### 2.2. Dietary Information Collection

Dietary intake data were collected using a 213-item general food frequency questionnaire (FFQ) developed for the Mongolian adult population. Consumption frequency was assessed using nine predefined categories ranging from never or <1 time per month to six or more times per day.

Foods were classified into eight food groups: milk and dairy products, grains and products, meat and meat products, fruits, vegetables, fats and oils, beverages, and ultra-processed foods (UPFs). From the above-mentioned food groups, selected categories were used to assess urban–nomadic dietary differences and their associations with *Bifidobacterium*. Standard portion sizes from the FFQ were applied for commonly consumed industrially processed foods, whereas portions of locally consumed traditional foods, including homemade dairy products, meat, and flour-based foods, were directly weighed using calibrated digital kitchen scales (±1 g precision) [29,30].

For each participant, the average daily intake (g/day) of every food item was calculated by converting reported consumption frequencies into daily equivalents and multiplying them by the corresponding portion weights, following methods previously applied in Mongolian dietary intake studies [25,31]. Foods consumed were converted to nutrients using previously established nutrient composition table [32,33,34].

### 2.3. DNA Extraction from Stool Samples

Genomic DNA was extracted from fecal samples preserved on Whatman FTA cards using the QIAamp PowerFecal Pro DNA Kit (QIAGEN, Hilden, Germany), following the manufacturer’s protocol with minor modifications. Approximately 250 mg of stool was applied to FTA cards, and one punched disc from each sample was transferred into a bead tube containing lysis buffer (Solution CD1). Following mechanical lysis, inhibitor removal, and silica membrane spin-column purification, DNA was eluted in 75 μL TE buffer (10 mM Tris, 0.1 mM EDTA) and stored at −20 °C until downstream analyses. This extraction protocol provides efficient recovery of microbial DNA from fecal samples while minimizing inhibitor carryover, making it suitable for shotgun metagenomic sequencing [35,36,37,38,39,40].

### 2.4. NGS Sequencing

Extracted DNA was prepared into DNA libraries following previously published protocols [35]. Briefly, DNA extracts underwent end repair and adapter ligation to generate double-stranded libraries, followed by limited-cycle amplification and dual indexing [40].

Sequencing libraries were pooled equimolarly and sequenced on an Illumina NovaSeq 6000 platform (Illumina, Inc., San Diego, CA, USA) (paired-end 150 bp configuration). The protocol was selected to ensure compatibility with downstream shotgun metagenomic sequencing and species-level taxonomic profiling.

Raw FASTQ files were demultiplexed and were processed using nf-core/eager v2.4.4 [41]. In brief, poly-G sequencing artefacts were removed using fastp v0.20.1 [42] before removing Illumina adapter sequences using AdapterRemoval v2.3.2 [43]. All read pairs that were longer than 30 bp were subsequently aligned against the human reference genome hs37d5 using BWA mem v0.7.17-r1188 [44] using default settings. All reads for which less than 100 bases could be aligned to the human reference genome were retained and randomly subsampled to 50 million sequences using seqtk v1.3. These sequences were taxonomically profiled using MetaPhlAn3 (v3.0.14; [45]) with the mpa_v30_CHOCOPhlAn_201901 database under default parameters. This approach relies on clade-specific marker genes to estimate relative abundances at species-level resolution. Relative abundance values were expressed as percentages of the total microbial community per sample. Across the Mongolian cohort, a total of 10 *Bifidobacterium* species were detected and retained for diversity, differential abundance, and association analyses.

### 2.5. Bioinformatics and Statistical Analyses

All downstream bioinformatic and statistical analyses were conducted in R (v4.3.0). Species-level relative abundance profiles derived from MetaPhlAn3 were used to calculate alpha diversity metrics of the *Bifidobacterium* community [45]. Shannon and Simpson diversity indices were computed using the vegan package based on species-level relative abundance data. Differences in alpha diversity between urban and nomadic groups and across geographic regions were assessed using non-parametric Kruskal–Wallis tests, and where appropriate, *p*-values were adjusted using the Benjamini–Hochberg false discovery rate (FDR) correction to account for multiple comparisons.

Beta diversity was calculated using Bray–Curtis dissimilarity and visualized by principal coordinates analysis (PCoA). Differences in community composition were tested using PERMANOVA with 9999 permutations, and R^2^ values were reported [46,47].

For global comparison, we used a sub-sample of the curated MetagenomicData R package [48], comprising 1286 healthy adults from 29 countries. Only non-confounded healthy adult cohorts, excluding individuals receiving antibiotics and those who were pregnant or lactating, with species-level taxonomic profiles generated using the same MetaPhlAn3 workflow and accompanied by sufficient metadata, were included in the analysis [49].

## 3. Results

### 3.1. Participant Characteristics

The present study included 100 adult participants (51 males and 49 females) aged 18–84 years who met the study eligibility criteria. Participants had no recent antibiotic or probiotic use and no severe gastrointestinal symptoms at the time of recruitment, as described in Section 2.1. Participants were categorized and analyzed according to geographical location, age, sex, and body mass index (BMI) (Table 1 and Table 2).

No significant differences were observed in age, sex, or BMI distribution between urban and nomadic participants (*p* > 0.05), nor among participants from different nomadic regions (*p* > 0.05; Table 1).

Significant differences were identified in dietary intake patterns across several food groups. Compared with the nomadic group, urban participants reported significantly higher daily intakes of meat, vegetables, and fruits (*p* < 0.001), whereas dairy consumption was substantially greater among nomadic participants (*p* < 0.001). Fruit intake differed markedly between groups, with urban participants consuming nearly six times more fruit than nomadic participants. Vegetable intake showed a similar pattern (*p* < 0.001), with urban participants consuming approximately 160–500 g/day compared with less than 80 g/day among nomadic participants. Grain-based food consumption also differed significantly between groups (*p* < 0.01), with greater variability among nomadic participants. Fat intake differed modestly but significantly between the two groups (*p* < 0.05), with higher variability among nomadic participants. Urban participants also reported significantly higher UPF intake compared with nomadic participants (*p* < 0.001; Table 2).

Further analysis within nomadic regions revealed pronounced regional heterogeneity in dietary intake (Table 2). Dairy consumption varied significantly across nomadic regions (*p* < 0.001), with the highest intake observed in Bulgan province (910 g/d), followed by Khuvsgul (560 g/d) and Gobi (310 g/d). Grain foods intake also differed significantly among nomadic regions (*p* < 0.05), with participants from Gobi (315 g/d) reporting the highest average intake. Fat group intake showed moderate but significant regional variation (*p* < 0.05), whereas meat, vegetable, and fruit consumption did not differ significantly across nomadic regions (*p* > 0.05).

Overall, demographic characteristics were comparable across study groups, whereas substantial regional differences were evident in dietary intake patterns, particularly for dairy, grain, and fat consumption. Dairy consumption exhibited the greatest regional variation, reflecting differences in traditional dietary practices across nomadic Mongolia. Given these distinct dietary patterns on regional level, next we examined the overall community structure and diversity of *Bifidobacterium* to characterize inter-individual and regional variability.

### 3.2. Community Structure and Diversity of Bifidobacterium

**Figure 1:**
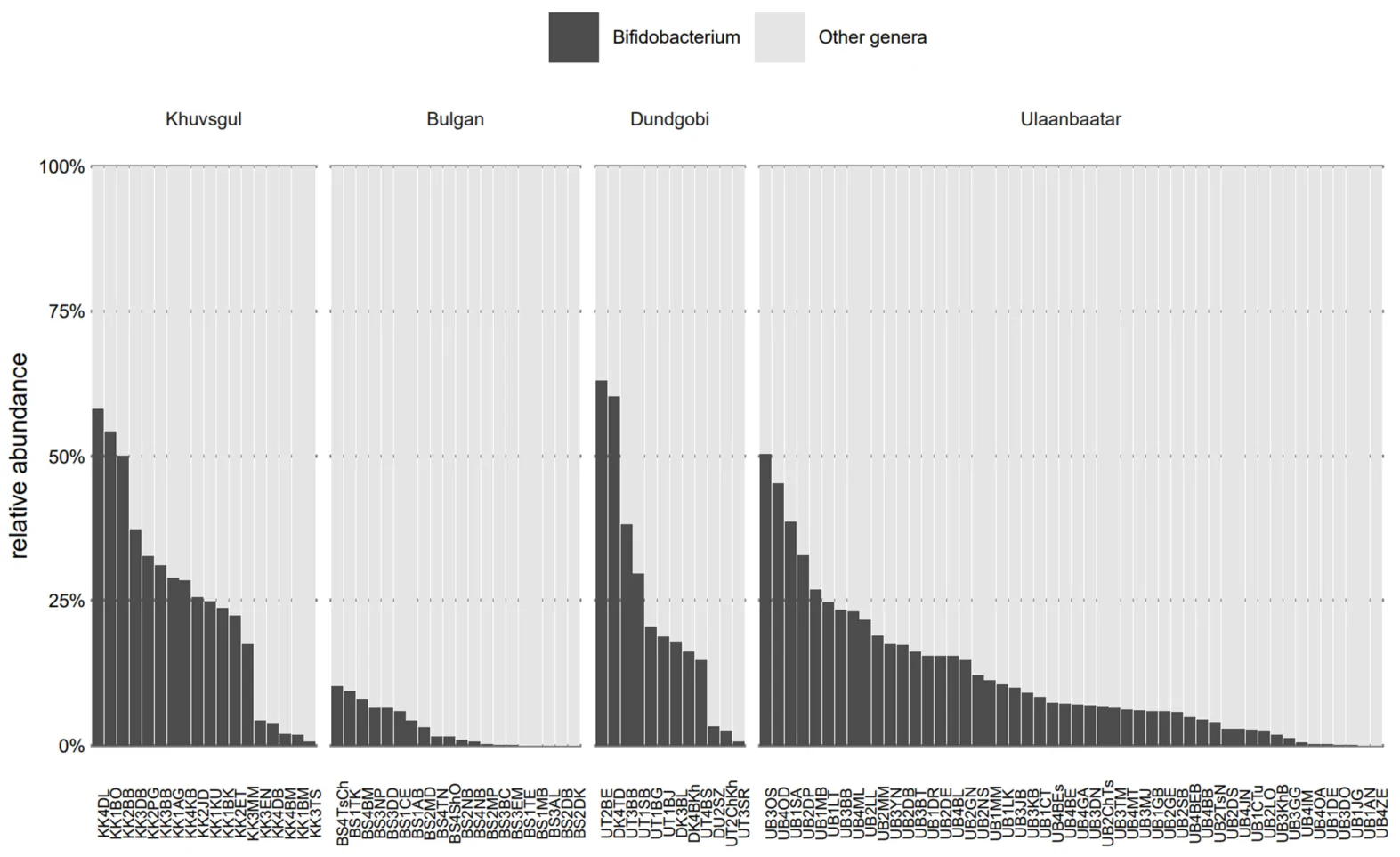
The relative abundance of the genus *Bifidobacterium* in the fecal samples of Mongolian adults (*n* = 100). Samples are ordered by decreasing relative abundance of *Bifidobacterium*.

The relative abundance of the *Bifidobacterium* genera in the gut microbiota were ordered by decreasing value to examine how *Bifidobacterium* community abundance varied within individuals and across geographic regions. Substantial differences were observed in the relative abundance of *Bifidobacterium* in both inter- and intra-groups of the study participants highlighting marked heterogeneity across individuals (Figure 1). The relative abundance of *Bifidobacterium* varied between 0.2% and 8% in Bulgan, while reaching 55–62% in Khuvsgul and Dundgobi, and 0–50% in urban populations.

**Figure 2:**
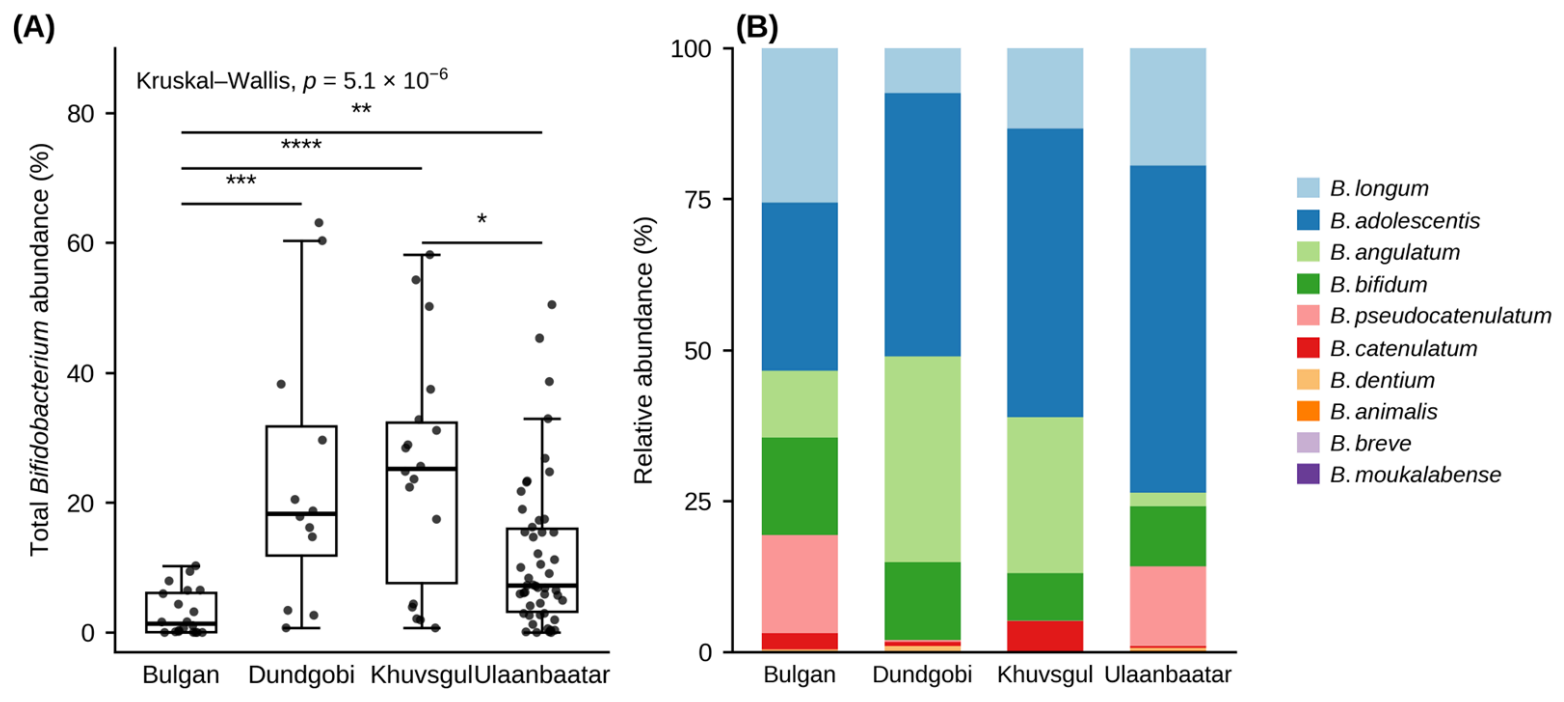
Regional variation in the relative abundance of predominant *Bifidobacterium* species across four Mongolian provinces. (**A**) Total relative abundance (%) of the predominant *Bifidobacterium* species in participants from Bulgan, Dundgobi, Khuvsgul, and Ulaanbaatar. Boxes represent the median and interquartile range (IQR), whiskers indicate 1.5 × IQR, and dots represent individual participants. Pairwise comparisons were performed using the Wilcoxon rank-sum test with Benjamini–Hochberg correction following a significant Kruskal–Wallis test. (**B**) Mean relative abundance (%) of the 10 predominant *Bifidobacterium* species in each province. Significance levels are indicated as *p* < 0.05 (*), *p* < 0.01 (**), *p* < 0.001 (***), and *p* < 0.0001 (****).

There was no significant difference in the total relative abundance of predominant *Bifidobacterium* species between nomadic and urban populations (Wilcoxon rank-sum test, *p* = 0.812). In contrast, the total relative abundance of predominant *Bifidobacterium* species differed significantly among the four geographical locations (Kruskal–Wallis test, *p* < 0.0001; Figure 2).

Beyond differences in overall relative abundance, regional variation in *Bifidobacterium* species diversity and community structure was further assessed using alpha diversity indices and Bray–Curtis-based ordination analysis.

**Figure 3:**
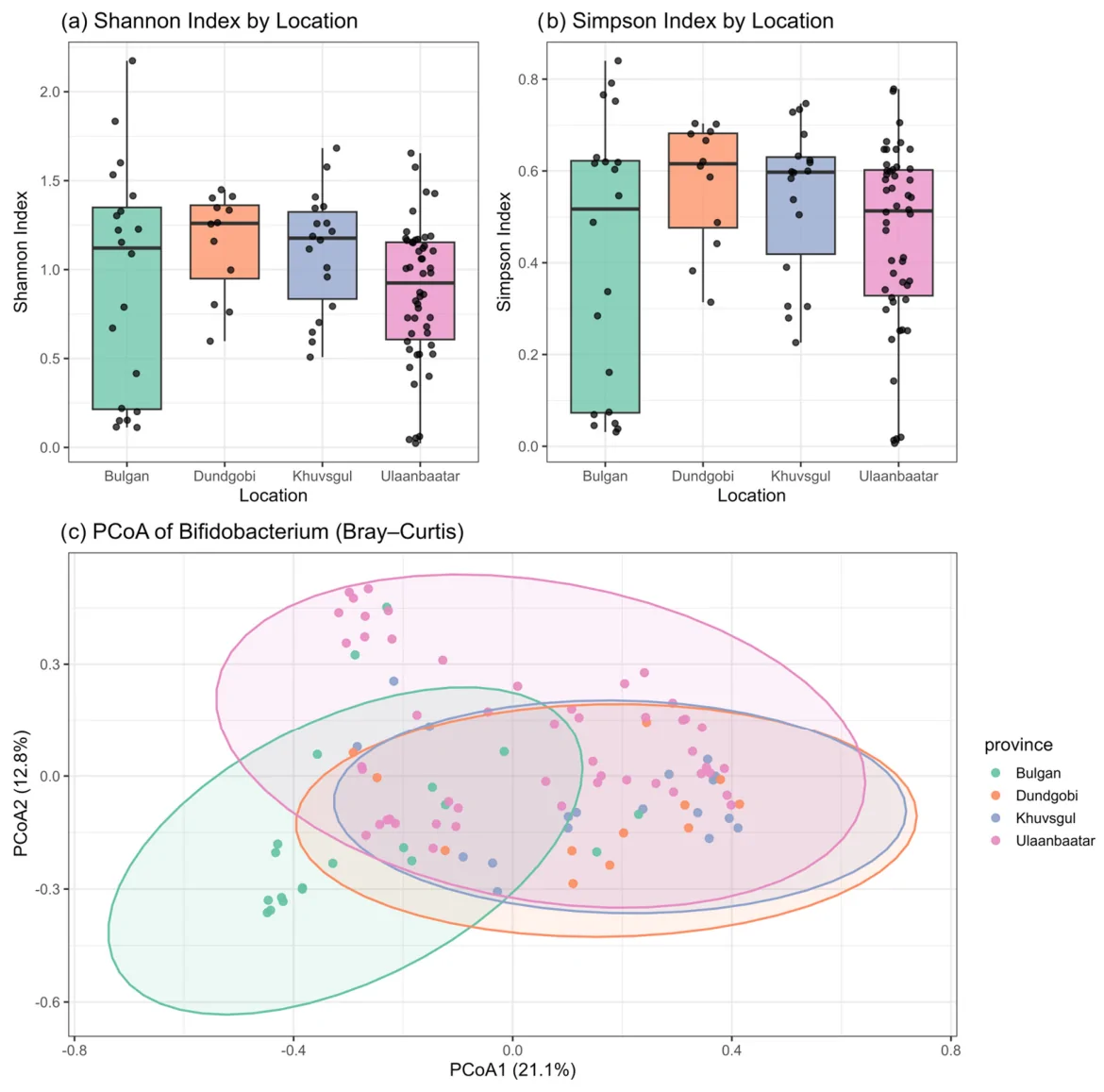
Alpha and beta diversity of the *Bifidobacterium* community across geographic regions. (**a**) Shannon diversity index calculated from species-level relative abundance of *Bifidobacterium*. (**b**) Simpson diversity index calculated from species-level relative abundance of *Bifidobacterium*. (**c**) Principal Coordinates Analysis (PCoA) of *Bifidobacterium* community composition based on Bray–Curtis dissimilarity. In panels (**a**,**b**), colored boxes indicate the geographic regions, and black points represent individual participants. In panel (**c**), colored points represent individual participants from each region, and shaded ellipses represent 95% confidence intervals for each region.

The Shannon diversity index of the *Bifidobacterium* community, calculated using species-level relative abundance data, was significantly higher in the nomadic regions (Khuvsgul and Dundgobi) than in the urban region (Ulaanbaatar) (*p* < 0.05), suggesting that traditional nomadic lifestyles may support greater *Bifidobacterium* species diversity. The Bulgan group exhibited lower *Bifidobacterium* diversity compared to other regions, though the difference was not statistically significant. Similarly, the Simpson diversity index of the *Bifidobacterium* community showed the same trend, with no statistically significant difference among regions (Kruskal–Wallis χ^2^ = 1.816, df = 3, *p* = 0.6115; Figure 3).

Beta diversity analysis based on Bray–Curtis dissimilarity revealed partial clustering of samples by geographic region in the PCoA ordination (Figure 3c). The first two principal coordinates explained 23.25% and 15.18% of the total variance, respectively. PERMANOVA analysis confirmed a significant effect of geographic region on *Bifidobacterium* community structure (R^2^ = 0.101, F = 3.50, *p* = 0.0001), indicating that approximately 10.1% of the observed variation was attributable to regional differences, whereas 89.9% reflected inter-individual variability.

Following the analysis of *Bifidobacterium* community diversity (Figure 3), we further examined the species-level relative abundance of *Bifidobacterium* to identify the taxa contributing most to regional and inter-individual variation.

**Figure 4:**
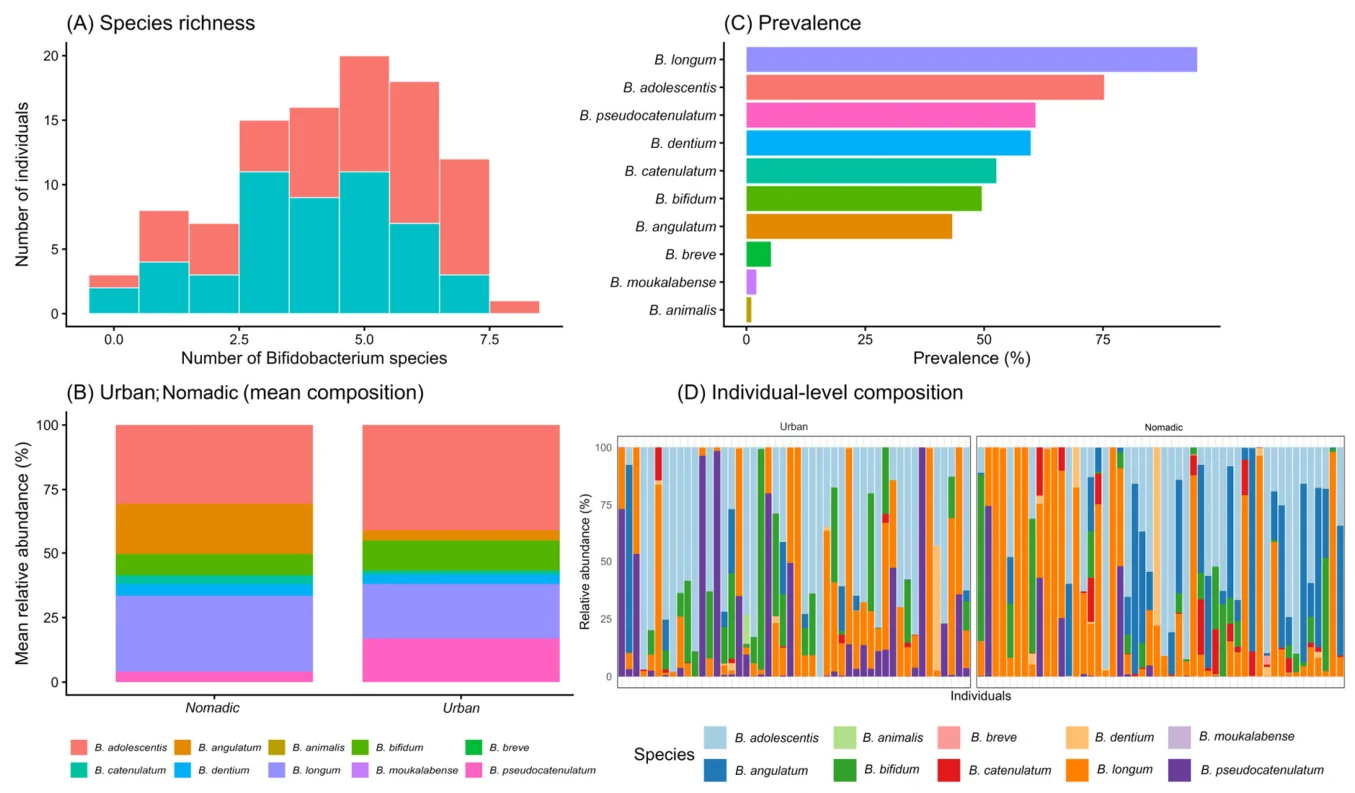
Diversity, prevalence, and composition of *Bifidobacterium* species in urban and nomadic adults. (**A**) Distribution of *Bifidobacterium* species richness per individual, where colors indicate population groups (red = urban, teal = nomadic). (**B**) Mean relative abundance (%) of *Bifidobacterium* species in urban and nomadic populations. (**C**) Prevalence (%) of *Bifidobacterium* species across all participants. (**D**) Individual-level relative abundance profiles of *Bifidobacterium* species across samples, with individuals ordered by population group (urban to nomadic). In panels (**B**–**D**), colors represent individual *Bifidobacterium* species as indicated in the respective legends.

The *Bifidobacterium* species richness ranged from 0 to 8 species per individual, with a median richness of 5 species (IQR: 3–6). The mean richness across all participants was 4.31 species per individual. Most individuals harbored between 3 and 6 *Bifidobacterium* species (Figure 4A).

Figure 4B illustrates the mean relative abundance of *Bifidobacterium* species in urban and nomadic participants. In the urban group, *B. adolescentis* was the most abundant species (40%), followed by *B. longum* (22%), *B. pseudocatenulatum* (16%), *B. bifidum* (12%), *B. angulatum* and *B. catenulatum* (4% each), *B. dentium* (3%), and *B. breve* (1%). In the nomadic group, *B. adolescentis* and *B. longum* each accounted for approximately 30% of the total *Bifidobacterium* composition, followed by *B. angulatum* (20%), *B. bifidum* (8%), *B. catenulatum* (5%), *B. dentium* (4%), *B. pseudocatenulatum* (3%), and *B. breve* (1%). *B. moukalabense* and *B. animalis* were detected at less than 1% relative abundance in both groups.

Among the detected *Bifidobacterium* species, *B. longum* showed the highest prevalence (95%), followed by *B. adolescentis* (75%). Moderate prevalence was observed for *B. pseudocatenulatum* (60%), *B. dentium* (58%), *B. catenulatum* (52%), and *B. bifidum* (50%). In contrast, *B. breve*, *B. moukalabense*, and *B. animalis* were rarely detected (Figure 4C).

*B. longum* exhibited a higher prevalence among nomadic participants (96%) compared to urban participants (88%). In contrast, *B. pseudocatenulatum* was more frequently detected in the urban group (72%). Meanwhile, *B. animalis* and *B. moukalabense* were identified in only a small proportion of participants (0–4%) (Figure 4D).

**Figure 5:**
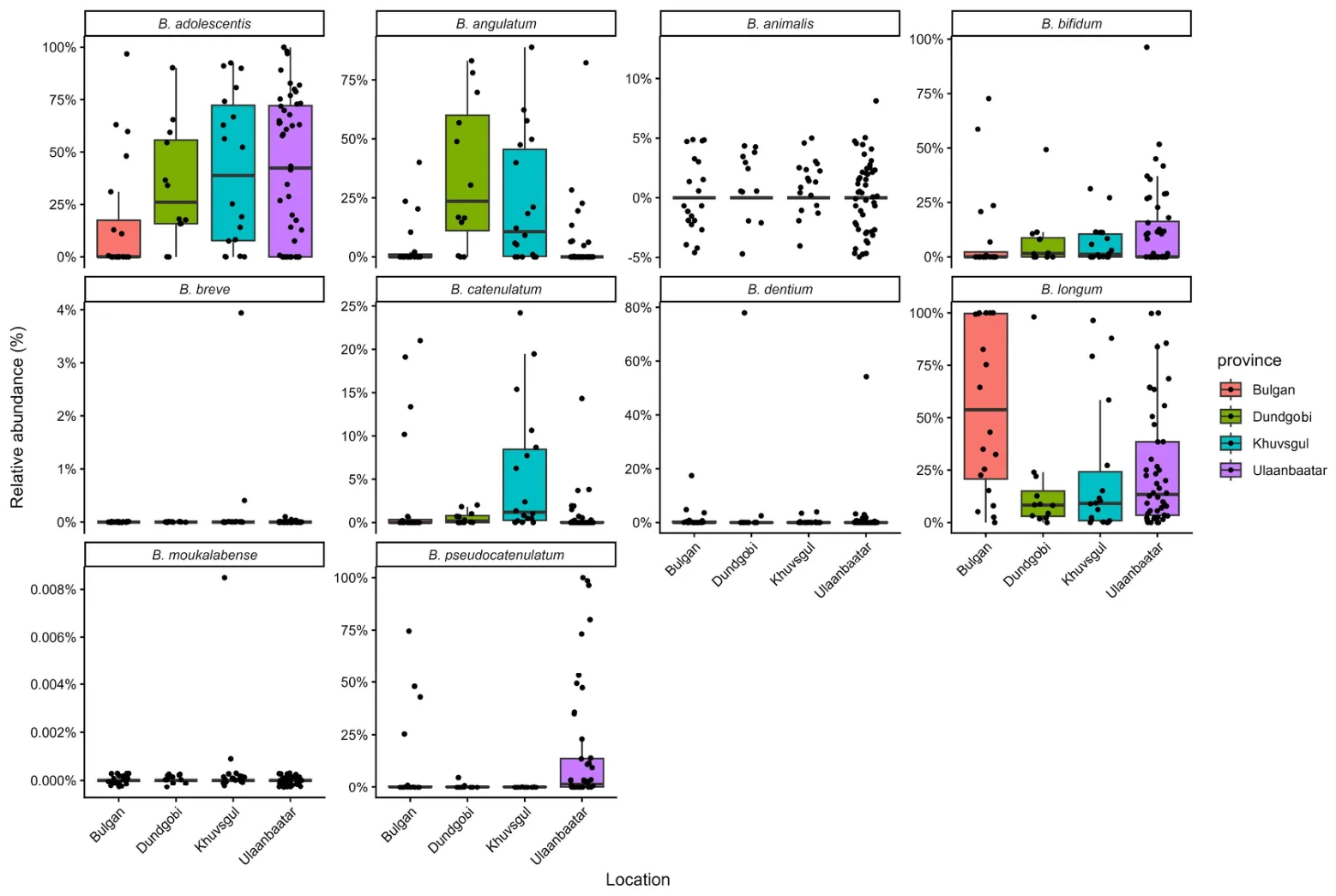
Relative abundance (%) of ten major *Bifidobacterium* species across four geographic regions of Mongolia (Khuvsgul, Bulgan, Dundgobi, and Ulaanbaatar). Boxplots display the distribution of species-level relative abundance based on MetaPhlAn-derived profiles. Black points represent individual participants. Statistical differences among locations were assessed using the Kruskal–Wallis test followed by Benjamini–Hochberg FDR correction.

Since clear differences were observed between urban and nomadic participants with distinct dietary consumption, we further explored regional variation in *Bifidobacterium* composition across four geographic locations in Mongolia (Figure 5).

In Ulaanbaatar, the *Bifidobacterium* community was predominantly characterized by *B. adolescentis* (median: 42.4%), followed by *B. longum* (13.4%) and *B. pseudocatenulatum* (1.54%). Other species, including *B. dentium*, *B. angulatum*, *B. bifidum*, *B. breve*, *B. catenulatum*, *B. animalis*, and *B. moukalabense*, were detected at relatively low median abundances. In contrast, nomadic populations showed relatively higher abundances of *B. longum* and *B. angulatum*, together with a more even distribution of *Bifidobacterium* species. Overall, nomadic communities exhibited greater species evenness, whereas urban individuals showed community profiles more strongly dominated by *B. adolescentis* and *B. pseudocatenulatum* (Figure 5).

Significant regional differences were observed in the composition of *Bifidobacterium* species. *B. angulatum* and *B. catenulatum* showed the greatest regional variation (FDR < 0.001), whereas *B. pseudocatenulatum*, *B. longum*, and *B. adolescentis* also differed significantly (FDR < 0.05). In contrast, *B. animalis*, *B. bifidum*, *B. breve*, and *B. dentium* remained relatively stable across regions.

The heatmap summarized the regional contribution to the total abundance of each *Bifidobacterium* species and was consistent with the individual-level compositional profiles shown in Figure 4D. The highest proportion of total *B. adolescentis* abundance was detected in samples from Ulaanbaatar (47.1%), whereas *B. pseudocatenulatum* was strongly concentrated in Ulaanbaatar, accounting for 85.7% of its total detected abundance. In contrast, *B. breve* (92.4%) and *B. catenulatum* (85.2%) were predominantly detected in Khuvsgul. More than half of the total *B. longum* abundance (54.1%) originated from Ulaanbaatar, whereas *B. angulatum* was mainly distributed in Khuvsgul (49.7%) and Dundgobi (41.9%). *B. dentium* was relatively more abundant in Dundgobi (48.4%) and Ulaanbaatar (39.8%). Among the low-abundance taxa, *B. animalis* was detected only in Ulaanbaatar, whereas *B. moukalabense* was identified exclusively in Khuvsgul.

**Figure 6:**
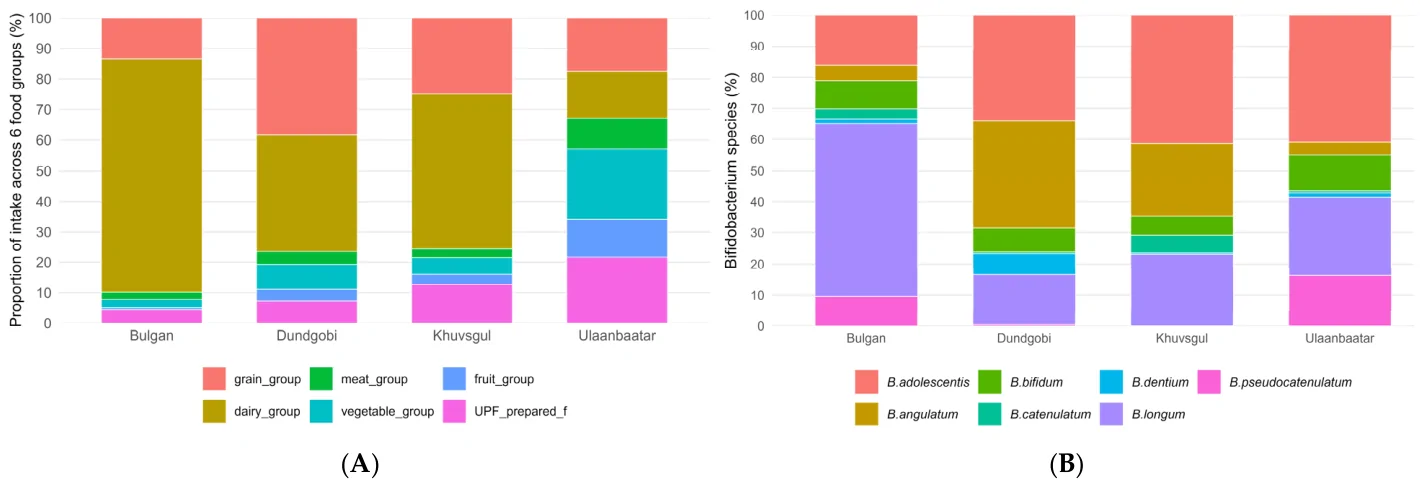
(**A**) Proportional contribution (%) of major dietary components, including grain, dairy, meat, fruit, vegetable, and ultra-processed food (UPF) groups, to total dietary intake across four provinces. (**B**) The relative abundance (%) of selected *Bifidobacterium* species across four provinces (Bulgan, Dundgobi, Khuvsgul, and Ulaanbaatar). In panels (**A**,**B**), colors represent the respective dietary food groups and *Bifidobacterium* species, as indicated in the legends.

To further explore the potential dietary factors underlying these regional differences, we examined the associations between dietary intake and *Bifidobacterium* species composition (Figure 6).

### 3.3. Associations Between Dietary Intake and Bifidobacterium Species

Dietary patterns were closely associated with regional differences in *Bifidobacterium* composition (Figure 6A,B). In Bulgan, dairy consumption was largely based on fermented mare’s milk (airag), whereas Khuvsgul populations consumed yak- and cow-based dairy products, including yogurt and traditional fermented foods. Dundgobi populations exhibited a more balanced dietary intake pattern, while urban populations relied more on industrially processed dairy products.

Notably, the Bulgan group showed the highest dominance of *B. longum* despite having the lowest total *Bifidobacterium* abundance. In both Khuvsgul and Dundgobi populations, the *Bifidobacterium* community was predominantly composed of *B. angulatum*, indicating a region-specific dominant species pattern.

The association between dietary intake and *Bifidobacterium* abundance was not uniform across regions, particularly for dairy consumption. For example, the Bulgan population exhibited relatively low *Bifidobacterium* levels despite having the highest fermented dairy intake.

Dairy products represented the major dietary component in Bulgan province, accounting for 85.2% of the total daily food intake (g/day), which was approximately 5.5-fold higher than that observed in Ulaanbaatar (15.5%). Interestingly, the most dominant *Bifidobacterium* species were *B. longum* followed by *B. adolescentis*, *B. bifidum* and *B. pseudocatenulatum*. In contrast, vegetable and fruit consumption together with ultra-processed food (UPF) was highest in Ulaanbaatar, but dairy consumption was the lowest among the four study regions. Meat intake was also notably higher in Ulaanbaatar than in the other provinces. With this dietary pattern, *B. adolescentis* was strongly dominant (55.5%) in urban population whereas *B. longum* (20.0%) and *B. pseudocatenulatum* (11.2%) were present at moderate levels (Figure 6A,B).

Participants from Dundgobi and Khuvsgul exhibited relatively mixed dietary patterns characterized by substantial contributions from both grain and fermented dairy products. In Dundgobi, grain and dairy products each accounted for approximately 38.5% of the total daily food intake (g/day). In Khuvsgul, fermented dairy products accounted for 44.3% of the total daily food intake (g/day), while grain products contributed 35.2%. Across all regions, *B. animalis*, *B. breve*, and *B. moukalabense* were detected only at very low abundances (<1%).

To further evaluate the relationship between dairy intake and *Bifidobacterium* composition, Spearman’s rank correlation analysis with Benjamini–Hochberg false discovery rate (FDR) correction was performed (Table S1). After FDR correction, significant associations were identified for three species. *B. angulatum* showed negative correlations with factory milk (ρ = −0.289, FDR = 0.028) and factory yogurt intake (ρ = −0.323, FDR = 0.014), but positive correlations with homemade yogurt (ρ = 0.293, FDR = 0.028) and total traditional dairy intake (ρ = 0.270, FDR = 0.046). *B. catenulatum* was positively associated with homemade yogurt (ρ = 0.331, FDR = 0.014), fermented dairy intake (ρ = 0.302, FDR = 0.025), and traditional dairy intake (ρ = 0.344, FDR = 0.013). In addition, *B. pseudocatenulatum* was positively correlated with factory milk intake (ρ = 0.346, FDR = 0.013). No significant associations were observed for *B. longum*, *B. adolescentis*, *B. bifidum*, or *B. dentium* after FDR correction (Table S1).

Taken together, these observations suggest that regional dietary differences antributable to dairy may be one of several factors associated with the distribution of key *Bifidobacterium* species among Mongolian adults.

### 3.4. Comparison with the Global Dataset

**Figure 7:**
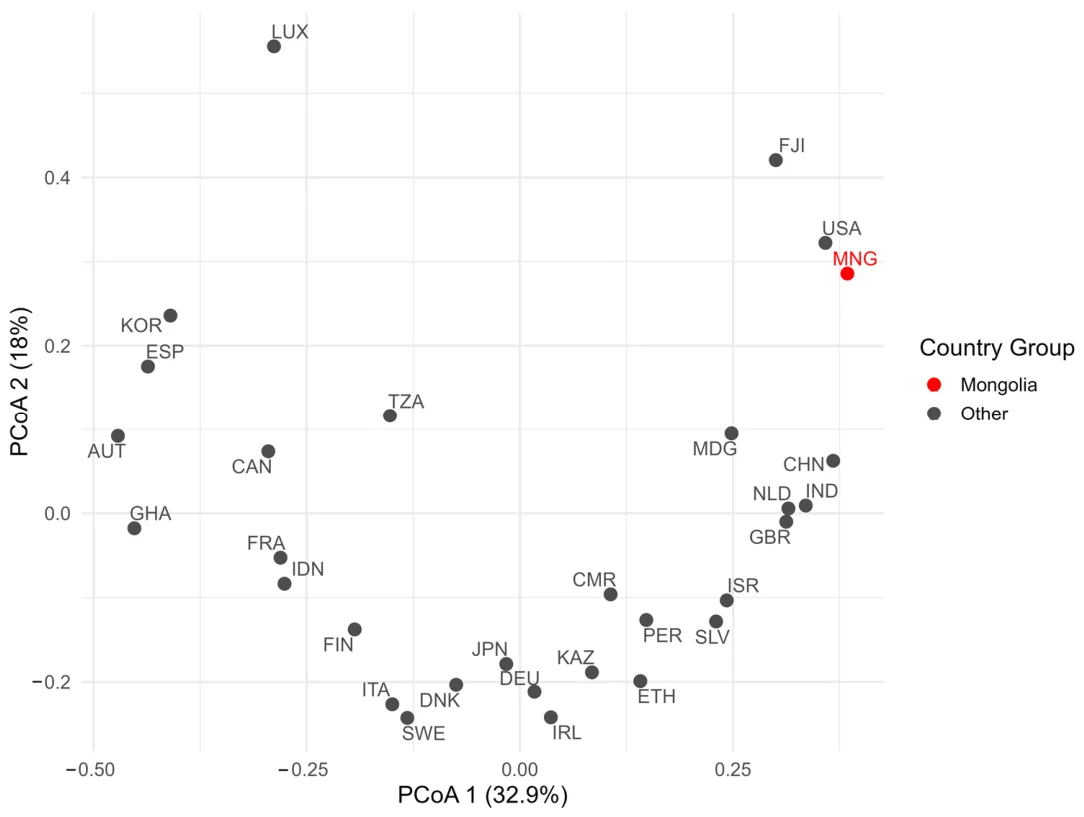
Comparative PCoA of *Bifidobacterium* Profiles Between Mongolia and International Cohorts. Principal Coordinates Analysis (PCoA) based on Bray–Curtis dissimilarity calculated from the relative abundances of *Bifidobacterium* species, showing cross-country variation in *Bifidobacterium* community composition. Each point represents the centroid of samples from a given country. The first two PCoA axes explained 32.9% (PCoA1) and 18.0% (PCoA2) of the total variance, respectively, together accounting for approximately 50.9% of the overall variation in community structure. The remaining variation was distributed across additional dimensions not represented in the two-dimensional plot. Mongolia (MNG) is highlighted in red, while grey points represent other countries. The spatial separation of Mongolia from several international cohorts suggests differences in *Bifidobacterium* community composition.

**Figure 8:**
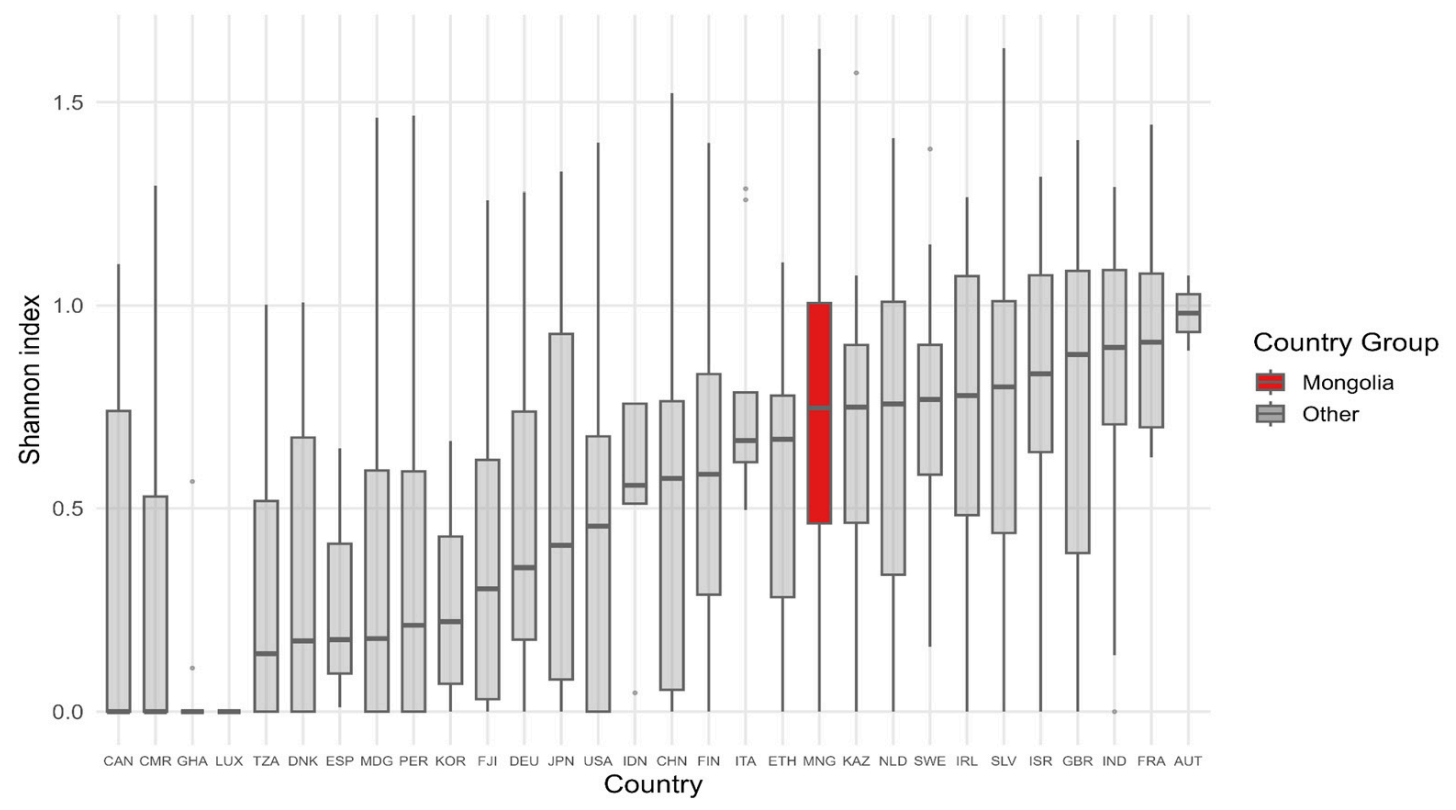
Global comparison of *Bifidobacterium* community structure and diversity based on species-level profiles. Boxplot comparison of Shannon diversity indices calculated based on *Bifidobacterium* species profiles across 30 countries, with Mongolia highlighted in red.

To place the findings into a broader global context, comparison of the *Bifidobacterium* species-level community structure of Mongolian adults with publicly available international datasets was performed. This genus-focused comparison allows to determine whether the patterns of *Bifidobacterium* composition and diversity observed in Mongolia are unique to this population or shared with other countries worldwide (Figure 7 and Figure 8).

PCoA based on Bray–Curtis dissimilarity revealed clear cross-country separation in *Bifidobacterium* community structure (Figure 7). The Mongolian centroid clustered separately from many international cohorts, suggesting a distinct community composition pattern. Interestingly, Mongolia appeared relatively closer to the USA and Fiji compared with several European and East Asian cohorts, although clear separation was still observed. PERMANOVA confirmed significant cross-country differences in *Bifidobacterium* community structure (R^2^ = 0.167, F = 6.86, *p* = 0.0001), indicating that geographic origin explained approximately 16.7% of the total variation in community composition. This suggests a moderate but biologically meaningful geographic effect on *Bifidobacterium* community structure across populations.

Across the 30 countries analyzed, Mongolia (MNG) exhibited relatively high Shannon diversity index values, indicating a high level of alpha diversity within the *Bifidobacterium* community and ranking among the higher-diversity cohorts globally (Figure 8). Compared with many other countries, the Mongolian cohort showed greater within-sample (*alpha*) diversity of *Bifidobacterium* species. The overall differences in Shannon diversity across countries were statistically significant (Kruskal–Wallis test: χ^2^ = 242.95, df = 30, *p* < 2.2 × 10^−16^).

Based on the distribution of the Shannon diversity index, the Mongolian cohort exhibited a relatively broad range of within-sample (*alpha*) diversity, comparable to that of the Netherlands and Ireland. The median Shannon diversity index was also similar to those observed in Kazakhstan, the Netherlands, Sweden, Ireland, and Slovenia, indicating that Mongolia belongs to the higher-diversity group among the 30 countries analyzed.

### 3.5. Phylogenetic Analysis

Phylogenetic analyses of the six predominant *Bifidobacterium* species revealed that Mongolian genomes were broadly distributed across multiple phylogenetic branches rather than forming distinct Mongolia-specific lineages (Supplementary Figures S1–S6). This pattern indicates substantial within-species genomic diversity among *Bifidobacterium* strains identified in Mongolian adults.

The phylogenetic tree of *B. adolescentis* showed that the 46 Mongolian genomes were distributed across multiple phylogenetic branches rather than forming a single Mongolia-specific lineage (Supplementary Figure S1), consistent with the overall pattern of substantial within-species genomic diversity.

Among the analyzed species, the phylogenetic tree of *B. angulatum* showed that the 19 Mongolian genomes were distributed across multiple phylogenetic branches (Supplementary Figure S2). While genomes such as MDC003_065 and MDC039_030 occupied relatively distant phylogenetic positions, MDC039_075 and MDC046_006 formed a closely related cluster.

Most *B. catenulatum* genomes identified in Mongolian participants originated from nomadic regions and were distributed across multiple phylogenetic branches, suggesting considerable genomic diversity among the nomadic isolates (Supplementary Figure S4).

## 4. Discussion

In the present study the association of diets, particularly the consumption of dairy, with the composition and diversity of human gut *Bifidobacterium* was elucidated with urban and nomadic population of Mongolia. The study populations represent traditional nomadic dietary practices coexisting with rapidly urbanizing food environments in Mongolia [25,26]. Also, the nomadic regions selected for this study represented distinct ecological zones of Mongolia with different animal milk sources and traditional dairy practices.

The dietary consumption of major food groups different significantly between the nomadic and urban groups with higher intake of fruits and vegetables, industrially processed foods and meat in participants from Ulaanbaatar versus the lower intake of these food groups in nomadic population. The grain group intake of nomads in Khuvsgul and Dundobi region were notably similar to urban population, however the nomadic people of Bulgan province were dominantly consuming the dairy products. These results were consistent with previously published findings in 2016–2020 on nutrition and dietary pattern of Mongolian population indicating the unchanged dietary habits in the recent years [33,50,51]. The dairy type differed significantly between the nomadic and urban groups with home-made milk products in nomadic population whereas the source of dairy in urban population were exclusively industrially processed ones. In Bulgan, the dairy consisted primarily of fermented mare’s milk product-airag, while the habitants in Khuvgul consumed the most the yak and cow milk yogurt along with other dairy products whereas the nomadic people in Dundgobi had fresh milk, yogurt and dried milk products made of camel, goat or cow milk.

Such distinct dietary patterns especially in terms of type of animal milk and fermented dairy products were associated with substantial shifts in both *Bifidobacterium* species composition and overall abundance. The most pronounced differences were observed across geographic regions of Mongolia. In Bulgan province, *Bifidobacterium* abundance remained consistently low, generally below 10%, whereas participants from Khuvsgul and Dundgobi frequently exhibited markedly higher abundances, reaching approximately 55–62% in some individuals. Urban participants from Ulaanbaatar exhibited the broadest abundance range (0–50%). Overall, the mean relative abundance of *Bifidobacterium* among Mongolian participants reached 13.6%, approaching the levels previously reported in Japanese populations (17.9 ± 15.2%), which are considered among the highest recorded worldwide [17,52,53].

Nomadic population of Mongolia were characterized by greater representation of species such as *B. breve* and *B. catenulatum*, whereas urban participants showed relatively higher contributions of *B. adolescentis*, *B. longum*, and *B. pseudocatenulatum*. Similar geographic variation in *Bifidobacterium* composition has been reported in other Asian populations, where traditional dietary practices were identified as major determinants of gut microbiota composition [24,25,54,55]. Compared with other international cohorts, Mongolia exhibited not only relatively high overall *Bifidobacterium* abundance but also substantial species-level diversity. The Mongolian gut microbiota was characterized by co-dominance of multiple species, particularly *B. adolescentis*, *B. longum*, *B. angulatum*, and *B. bifidum*, whereas several other countries showed stronger dominance by fewer taxa. Previous studies in Japanese populations reported that adult gut microbiota are typically dominated by *B. adolescentis* and *B. catenulatum*, whereas *B. breve* is more commonly associated with younger age groups [56]. Interestingly, Mongolian nomadic participants, particularly from Khuvsgul province, maintained relatively high abundances of *B. breve* and *B. catenulatum*. Although *B. breve* is typically considered an infant-associated species and generally declines with age, it was relatively enriched among adult nomadic Mongolian participants, particularly those from Khuvsgul [57,58,59]. These species-level differences were further reflected in community diversity metrics. Nomadic populations from Khuvsgul and Dundgobi provinces, exhibited significantly higher Shannon diversity indices compared with urban participants, indicating greater species heterogeneity within traditional nomadic microbiomes. In contrast, urban participants showed comparatively lower diversity together with stronger dominance of a limited number of taxa, particularly *B. adolescentis* and *B. pseudocatenulatum*.

Previous studies have suggested a positive association between fermented dairy consumption and *Bifidobacterium* abundance [7,26,60]. However, this relationship was not consistently observed in our study. Despite maintaining the most dairy-rich dietary pattern, participants from Bulgan province exhibited relatively low overall *Bifidobacterium* abundance compared with other regions. One possible explanation is that the Bulgan population relied heavily on fermented mare’s milk (airag) during summer months, often at the expense of dietary diversity and plant-derived fiber intake. Excessive dependence on a limited range of fermented dairy products may reduce the diversity of fermentable carbohydrate substrates available for *Bifidobacterium* species. In addition, spontaneous yeast- and LAB-associated fermentation during airag production may create ecological conditions that selectively favor competing microbial taxa while limiting the competitiveness of certain bifidobacterial populations [61,62,63]. LAB rapidly ferments lactose into lactic acid, potentially reducing carbohydrate availability for *Bifidobacterium*, whereas yeasts may further alter gut conditions through ethanol and organic acid production [24,64,65,66,67]. Together, these processes may contribute to reduced bifidobacterial diversity despite high fermented dairy intake. Compared with cow’s milk, mare’s milk contains higher concentrations of oligosaccharides, whereas lactose availability becomes markedly reduced following fermentation. Such conditions may selectively support more resilient species such as *B. longum* [68,69] since this species is among the most acid- and stress-tolerant members of the genus and can utilize a broader range of carbohydrates under nutrient-limited conditions. The central presence of *B. longum* among other species in Bulgan population suggests that this species may be more resilient under conditions where factors supporting bifidobacterial growth are limited [70,71,72]. In addition, mare’s milk contains relatively high concentrations of whey proteins and bioactive compounds that may further influence microbial ecology. Collectively, these factors—including acidic conditions, microbial competition, reduced carbohydrate availability, ethanol exposure, and seasonal dietary monotony—likely contribute to the pronounced decline in *Bifidobacterium* diversity observed in this population [25,33,73]. The discrepancy observed in Bulgan suggests that fermented dairy intake alone may not be sufficient to promote *Bifidobacterium* colonization. Other factors such as the type of fermented dairy products, frequency of their consumption and possibly the milk source of animals may play a critical role in shaping this bacterial species [74].

In contrast to Bulgan, participants from Dundgobi and Khuvsgul exhibited more similar dietary structures accompanied by relatively comparable *Bifidobacterium* community compositions. The strong geographic gradient observed within Mongolia—characterized by enrichment of *B. adolescentis* and *B. angulatum* in Khuvsgul and Dundgobi—highlights pronounced regional structuring of the *Bifidobacterium* community. These patterns may reflect the combined ecological influence of traditional dairy-centered diets and household-prepared fermented dairy foods commonly consumed in nomadic Mongolian populations. Previous dietary surveys, together with our findings, indicate that residents of Khuvsgul consume relatively high amounts of dairy products, root vegetables, and traditional flour-based foods [51,75]. Roots and wheat flour foods provide inulin, β-glucans, arabinoxylans, and other complex polysaccharides that are known to be utilized by *B. adolescentis* and *B. angulatum* [76,77]. These species are well recognized for its capacity to ferment resistant starches and plant-derived oligosaccharides, producing short-chain fatty acids with potential health benefits [4,53,78]. Elevated intake of fiber-rich plant foods together with LAB-rich fermented dairy products may contribute to higher abundances of these species, consistent with observations from other populations [16,18,53].

Traditionally fermented dairy products harbor naturally assembled microbial communities that develop through spontaneous fermentation under local environmental conditions. Unlike industrial dairy products, which are produced using a small number of well-defined commercial starter cultures, traditionally fermented milk contains complex and dynamic associations of lactic acid bacteria, yeasts, and other environmental microorganisms [17,18]. This greater microbial diversity may influence the intestinal ecosystem by providing a wider range of metabolic activities and ecological interactions that indirectly support members of the genus *Bifidobacterium* [16,17]. Although overall grain intake in Khuvsgul was comparable to that in Ulaanbaatar, the types of grain products consumed differed between regions, with Khuvsgul participants consuming more traditional grain-based foods and urban participants consuming more refined and ultra-processed grain products. These dietary differences may partly explain the higher abundance of *B. angulatum* observed in Khuvsgul [24,54,55]. This species is relatively rare in many Western cohorts [60,79,80]. Notably, in our study, industrially produced dairy consumption was negatively associated with *B. angulatum* but positively associated with *B. pseudocatenulatum*. Commercial dairy products are typically pasteurized and standardized, resulting in lower microbial diversity than traditional homemade fermented products. Furthermore, industrial processing alters the microbial community naturally present in raw milk, potentially reducing opportunities for microbial interactions that support certain bifidobacterial species [81,82,83]. Also, lower dairy product consumption and the resulting reduced lactose intake among urban participants may partly explain the lower relative abundance of *B. longum*. *B. catenulatum*, an adult-associated species capable of degrading diverse plant polysaccharides, was more frequently detected in Khuvsgul participants, consistent with its association with traditional fiber-rich diets reported in previous studies of human populations [84,85]. Although urban participants consumed greater amounts of fruits and vegetables, they also exhibited substantially higher meat and ultra-processed food intake compared with nomadic populations. Excessive reliance on animal protein and Westernized dietary patterns has previously been associated with reduced microbial diversity and lower abundance of beneficial saccharolytic taxa in multiple populations [7,60,80]. In this context, the comparatively lower *Bifidobacterium* diversity observed in urban participants may reflect the combined effects of high meat intake, reduce dietary complexity, and increase dietary industrialization. Conversely, increased consumption of ultra-processed foods has been linked to reduced microbial diversity and alterations in metabolic pathways associated with inflammation and chronic disease risk [26,86,87]. These findings also suggest that the effects of dairy consumption on the gut microbiota depend not only on the quantity consumed but also on the degree of processing and fermentation.

To further contextualize the study findings, we compared the Mongolian *Bifidobacterium* profile with previously reported international cohorts. The Mongolian cohort was predominantly characterized by *B. adolescentis*, followed by *B. angulatum* and *B. longum*. Comparative species-level profiling suggested that the overall *Bifidobacterium* composition of Mongolian adults was more similar to cohorts from Slovenia, India, Ethiopia, and Kazakhstan than to several East Asian populations. This pattern may be associated with the coexistence of traditional dietary practices and ongoing nutrition transition in Mongolia [2,4,25]. Species-level phylogenetic trees revealed that Mongolian genomes were broadly distributed across different phylogenetic branches rather than forming a single country-specific cluster, suggesting considerable intra-species genomic diversity (Supplementary Figures S1–S6).

Overall, the Mongolian cohort exhibited a distinct *Bifidobacterium* community structure characterized by region-specific ecological signatures linked to traditional dietary practices and ongoing nutritional transition [25,26]. Together, these findings indicate that culturally specific dietary practices, particularly fermented dairy consumption, may contribute substantially to sustaining *Bifidobacterium* abundance not only on the prevalence but also on species level in the Mongolian gut microbiota, highlighting the importance of considering regional dietary patterns when interpreting cross-population microbiome differences [16,53,88]. This study provides a detailed characterization of *Bifidobacterium* community structure in Mongolian adults and highlights the influence of traditional dietary practices including dairy fermentation with various animal milk on microbial composition. Previous cross-sectional study of Mongolian populations found that nomadic herders consume significantly more animal-source foods, including dairy products, than urban residents, whereas beneficial microbes, such as lactic acid bacteria and *Bifidobacterium* spp., were enriched in in this population [33]. Although high intake of animal-derived foods is often associated with reduced microbial diversity [26], the frequent consumption of fermented dairy products in nomadic populations may partially explain the enrichment of beneficial bacteria, such as LAB and *Bifidobacterium* [16,18,88]. Future studies integrating metagenomics and metabolomics approaches are warranted to further elucidate the functional capacity of these microbial communities, particularly in relation to fermented dairy consumption, effect of different animal milks and microbial resilience in low-fiber dietary environments. Several limitations should be acknowledged. Dietary intake was assessed in summer season only using food frequency questionnaires, which may not fully capture seasonal variation or portion size accuracy. In addition, the cross-sectional design precludes causal inference, and observed associations should be interpreted as hypothesis-generating. Although the study included 100 participants, the sample size does not fully represent the diversity of the Mongolian population, and participants were recruited from four selected regions with distinct dairying practices rather than from all geographic regions of the country. In particular, recruitment in Dundgobi Province was constrained by the very low population density and the large geographic distances between nomadic herder households, resulting in a relatively small regional sample size. Consequently, statistical power for some regional comparisons may have been limited, increasing the possibility of Type II errors. However, given the substantial cost, computational resources, and bioinformatics expertise required for shotgun metagenomic sequencing, this sample size is comparable to that of many microbiome studies and was considered sufficient to characterize regional variation in gut *Bifidobacterium* diversity. Furthermore, the inclusion of four geographically and nutritionally distinct regions provides valuable baseline data for the design of future large-scale, nationally representative studies of the Mongolian gut microbiome. Despite these limitations, our findings demonstrate that Mongolia’s unique combination of region-specific traditional dairy consumption and limited exposure to ultra-processed foods plays a central role in shaping a distinctive *Bifidobacterium* community structure in adults.

## 5. Conclusions

This study provides the first detailed, regionally stratified characterization of gut *Bifidobacterium* communities in Mongolian adults and identifies distinct microbial patterns associated with traditional dietary practices and contrasting lifestyles. Overall, Mongolian participants exhibited relatively high *Bifidobacterium* diversity, with nomadic regions, particularly Khuvsgul and Dundgobi, showing pronounced enrichment of *B. adolescentis* and *B. angulatum*. These patterns were consistent with high consumption of fermented dairy products, root vegetables, and unrefined carbohydrates that provide fermentable substrates, including both plant-derived polysaccharides and dairy-associated carbohydrates. In contrast, urban residents exhibited elevated levels of *B. pseudocatenulatum*, potentially reflecting greater intake of starch-rich and processed foods. Species-level correlation analyses further demonstrated significant associations between specific dairy products and the relative abundance of selected *Bifidobacterium* species after false discovery rate correction. These findings support an association between dietary practices and variation in *Bifidobacterium* community composition in Mongolian adults. Together, these findings suggest that non-industrialized dietary practices are associated with distinct *Bifidobacterium* community structures and contribute to Mongolia’s unique microbial profile in a global context. Future studies incorporating functional metagenomics and longitudinal dietary assessment are warranted to clarify the health implications of these region-specific microbial signatures.

## Figures and Tables

**Table 1:** Demographic and Anthropometric Characteristics of the Study Participants.

Characteristics	Urban (*n* = 50)	Nomadic (*n* = 50)	*p*-Value ᵃ	Khuvsgul (*n* = 18)	Bulgan (*n* = 20)	Gobi (*n* = 12)	*p*-Value ᵇ
	Median (IQR) or *n* (%)			Median (IQR) or *n* (%)			
Age (years)	41.0 (32.0–51)	44.0 (34.0–53.0)	>0.05	44.0 (35.0–52.0)	41.0 (32.0–49.0)	48.0 (38.0–58.0)	>0.05
Sex (male), *n* (%)	24 (48%)	27 (54%)	>0.05	9 (50%)	12 (60%)	6 (50%)	>0.05
BMI (kg/m^2^)	26.0 (22.8–29.4)	27.1 (23.6–30.2)	>0.05	26.1 (22.9–29.0)	27.4 (23.8–30.6)	25.8 (22.0–29.9)	>0.05

ᵃ *p*-values were calculated using the Mann–Whitney U test between urban and nomadic groups. ᵇ *p*-values were calculated using the Kruskal–Wallis test among nomadic regions.

**Table 2:** Daily Food Group Consumption According to Residential Area, g/day.

Characteristics	Urban (*n* = 50)	Nomadic (*n* = 50)	*p*-Value ᵃ	Nomadic (*n* = 50)	Khuvsgul (*n* = 18)	Bulgan (*n* = 20)	Gobi (*n* = 12)	*p*-Value ᵇ
	Median (IQR) or *n* (%)			Characteristics	Median (IQR) or *n* (%)			
Grain group	310.0 (210.0–420.0)	355.0 (240.0–510.0)	<0.01 *	Grain group	260.0 (180.0–360.0)	245.0 (160.0–390.0)	315.0 (210.0–470.0)	<0.05 *
Meat group	110.0 (60.0–180.0)	35.0 (15.0–65.0)	<0.001 *	Meat group	28.0 (18.0–42.0)	35.0 (20.0–60.0)	32.0 (22.0–55.0)	>0.05
Dairy group	180.0 (90.0–420.0)	1420.0 (680.0–2600.0)	<0.001 *	Dairy group	560.0 (320.0–780.0)	910.0 (600.0–1500.0)	310.0 (180.0–520.0)	<0.001 *
Vegetables group	300.0 (160.0–520.0)	45.0 (25.0–80.0)	<0.001 *	Vegetables group	55.0 (30.0–80.0)	42.0 (25.0–65.0)	60.0 (35.0–95.0)	>0.05
Fruits group	150.0 (70.0–320.0)	25.0 (10.0–55.0)	<0.001 *	Fruits group	38.0 (20.0–60.0)	12.0 (5.0–20.0)	30.0 (15.0–50.0)	>0.05
Fat/oil group	95.0 (70.0–120.0)	105.0 (80.0–145.0)	<0.05 *	Fat/oil group	110.0 (85.0–135.0)	125.0 (95.0–155.0)	70.0 (55.0–95.0)	<0.05 *
UPF group	199.9 (94.7–498.9)	61.4 (34.8–141.3)	<0.001 *	UPF group	93.8 (54.4–229.8)	56.9 (34.2–126.2)	0 (0-0)	>0.05

ᵃ *p*-values were calculated using the Mann–Whitney U test. ᵇ *p*-values were calculated using the Kruskal–Wallis test among nomadic regions. * Statistically significant at *p* < 0.05.

## Data Availability

The data presented in this study are not publicly available due to ethical and privacy restrictions related to human subjects. De-identified individual-level data may be made available from the corresponding author upon reasonable request and with approval from the relevant ethics committees.

## Supplementary Materials

The following supporting information can be downloaded at: https://www.mdpi.com/article/10.3390/nu18172816/s1, Figure S1. Maximum-likelihood phylogenetic tree of *Bifidobacterium adolescentis*. The tree was constructed using 1518 core genes from 2623 publicly available genomes together with 46 genomes identified in Mongolian participants. Red circles indicate Mongolian genomes, and circle size corresponds to the number of genomes detected. Numbers at the nodes represent bootstrap support values (%). The phylogenetic analysis included 946 core genes and 572 outgroup genes; Figure S2. Maximum-likelihood phylogenetic tree of *B. angulatum*. The tree was constructed using 898 core genes from 71 publicly available genomes together with 19 genomes identified in Mongolian participants. Red circles indicate Mongolian genomes, and circle size corresponds to the number of genomes detected. Numbers at the nodes represent bootstrap support values (%). The phylogenetic analysis included 802 core genes and 96 outgroup genes; Figure S3. Maximum-likelihood phylogenetic tree of *B. bifidum*. The tree was constructed using 1352 core genes from 1300 publicly available genomes together with 41 genomes identified in Mongolian participants. Red circles indicate Mongolian genomes, and circle size corresponds to the number of genomes detected. Numbers at the nodes represent bootstrap support values (%). The phylogenetic analysis included 1248 core genes and 104 outgroup genes; Figure S4. Maximum-likelihood phylogenetic tree of *B. catenulatum*. The tree was constructed using 1996 core genes from 314 publicly available genomes together with 23 genomes identified in Mongolian participants. Red circles indicate Mongolian genomes, and circle size corresponds to the number of genomes detected. Numbers at the nodes represent bootstrap support values (%). The phylogenetic analysis included 1055 core genes and 941 outgroup genes; Figure S5. Maximum-likelihood phylogenetic tree of *B. longum*. The tree was constructed using 1006 core genes from 203 publicly available genomes together with 65 genomes identified in Mongolian participants. Red circles indicate Mongolian genomes, and circle size corresponds to the number of genomes detected. Numbers at the nodes represent bootstrap support values (%). The phylogenetic analysis included 924 core genes and 82 outgroup genes; Figure S6. Maximum-likelihood phylogenetic tree of *B. pseudocatenulatum*. The tree was constructed using 1665 core genes from 1324 publicly available genomes together with 20 genomes identified in Mongolian participants. Red circles indicate Mongolian genomes, and circle size corresponds to the number of genomes detected. Numbers at the nodes represent bootstrap support values (%). The phylogenetic analysis included 908 core genes and 757 outgroup genes; Table S1. Spearman correlation analysis between dairy intake variables and the relative abundance of *Bifidobacterium* species. *p* values were adjusted using the Benjamini–Hochberg false discovery rate (FDR) method.

## Abbreviations

The following abbreviations are used in this manuscript:

BMI	Body Mass Index
MNUMS	Mongolian National University of Medical Sciences
PCR	Polymerase Chain Reaction
SCFA	Short-Chain Fatty Acids
SD	Standard Deviation
SPSS	Statistical Package for the Social Sciences
R	R Statistical Computing Environment
UB	Ulaanbaatar
LAB	Lactic acid bacteria
FDR	Benjamini–Hochberg false discovery rate
FFQ	Food Frequency Questionnaire
UPF	Ultra-Processed Foods
FTA	Flinders Technology Associates card
NGS	Next-Generation Sequencing
PcoA	Principal Coordinates Analysis
PERMANOVA	Permutational Multivariate Analysis of Variance
α-diversity	Alpha diversity
β-diversity	Beta diversity
FC	Fold Change
Log2FC	Log2 Fold Change
SE	Standard Error
