# Supplementary material for: Distinctive Patterns of Gut Bifidobacterium Diversity in Mongolian Adults: Regional Variation and Dairy Intake Associations
Source: Nutrients. 2026 Aug 28;18(17):2816. doi: 10.3390/nu18172816 (PMC13567504; doi:10.3390/nu18172816)
Supplement: Supplementary file 1 [file nutrients-18-02816-s001.zip › nutrients-4464995-supplementary.pdf]

nutrients

MDPI

B. adolescentis: Phylogenetic tree including 46 Mongolian genomes

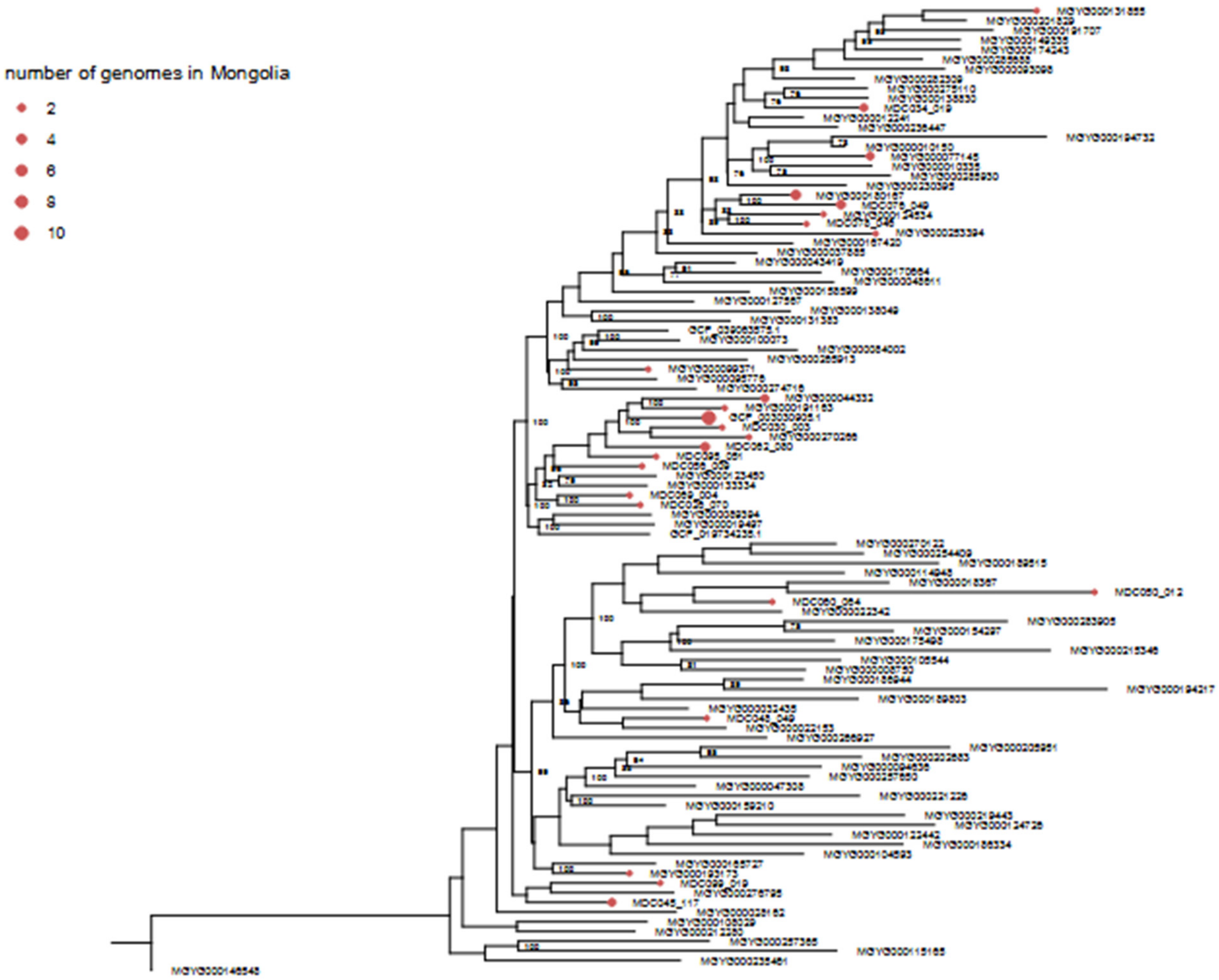

Figure S1. Maximum-likelihood phylogenetic tree of *Bifidobacterium adolescentis*. The tree was constructed using 1,518 core genes from 2,623 publicly available genomes together with 46 genomes identified in Mongolian participants. Red circles indicate Mongolian genomes, and circle size corresponds to the number of genomes detected. Numbers at the nodes represent bootstrap support values (%). The phylogenetic analysis included 946 core genes and 572 outgroup genes.

B. angulatum: Phylogenetic tree including 19 Mongolian genomes

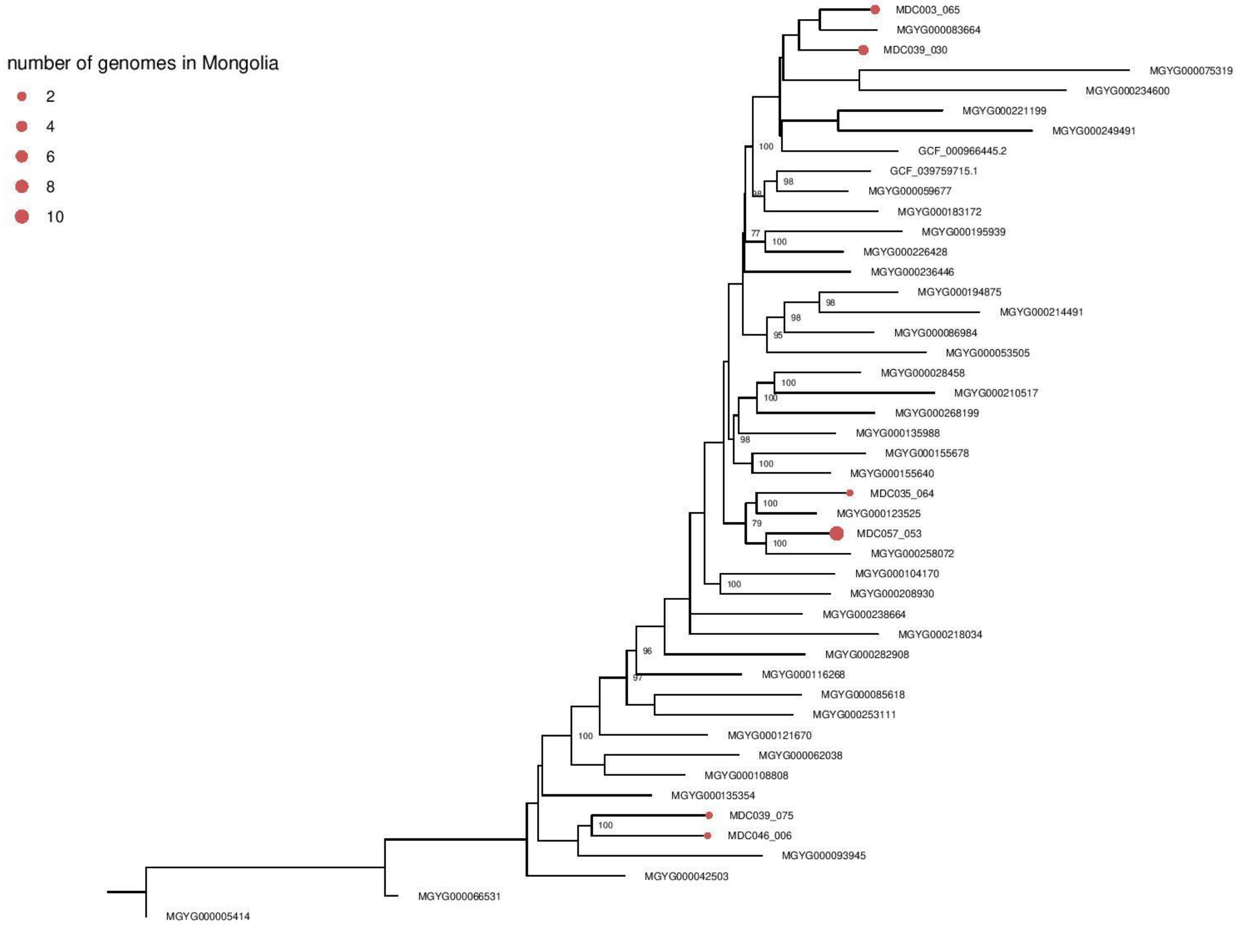

Figure S2. Maximum-likelihood phylogenetic tree of *B. angulatum*. The tree was constructed using 898 core genes from 71 publicly available genomes together with 19 genomes identified in Mongolian participants. Red circles indicate Mongolian genomes, and circle size corresponds to the number of genomes detected. Numbers at the nodes represent bootstrap support values (%). The phylogenetic analysis included 802 core genes and 96 outgroup genes.

Bifidobacterium bifidum: Phylogenetic tree including 41 Mongolian genomes

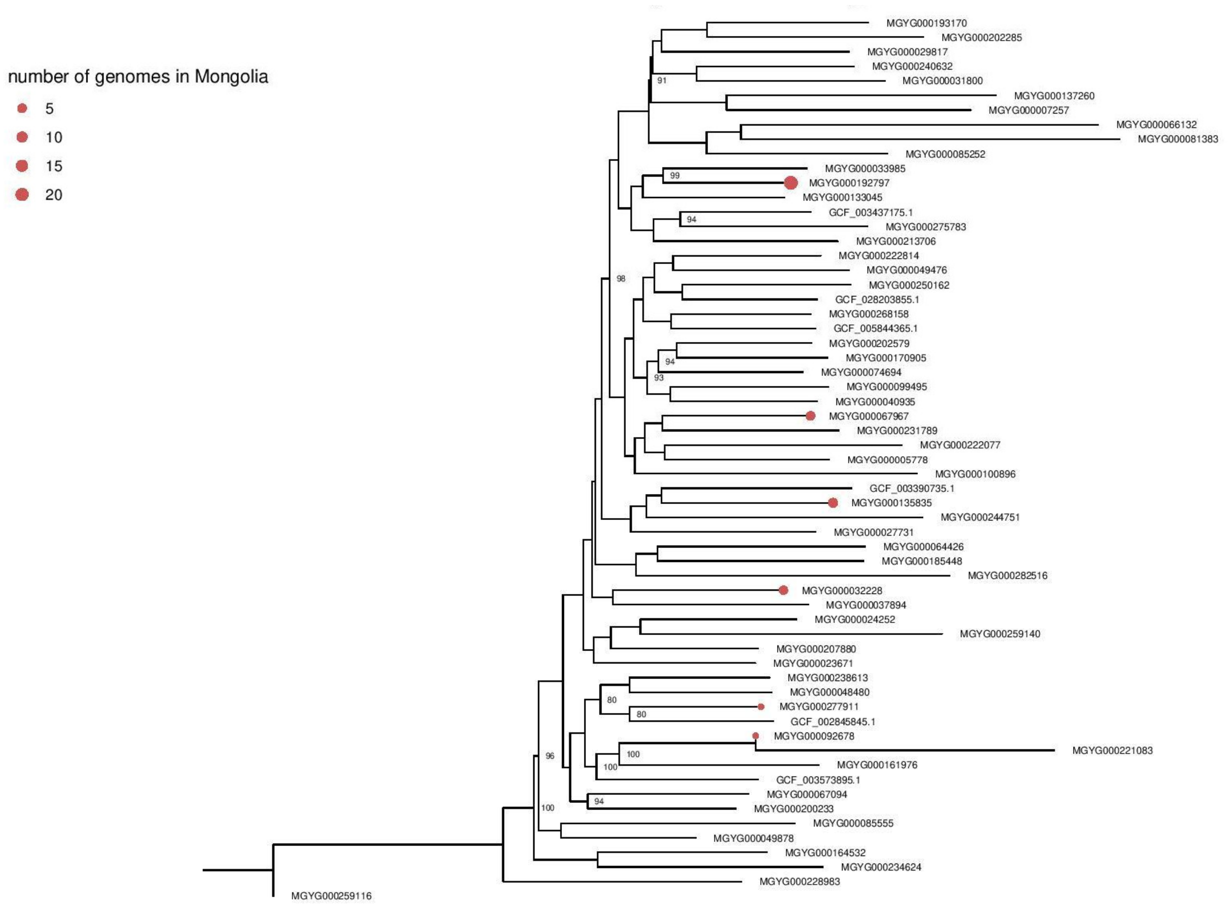

Figure S3. Maximum-likelihood phylogenetic tree of *B. bifidum*. The tree was constructed using 1,352 core genes from 1,300 publicly available genomes together with 41 genomes identified in Mongolian participants. Red circles indicate Mongolian genomes, and circle size corresponds to the number of genomes detected. Numbers at the nodes represent bootstrap support values (%). The phylogenetic analysis included 1,248 core genes and 104 outgroup genes.

***Bifidobacterium catenulatum*: Phylogenetic tree including 23 Mongolian genomes**

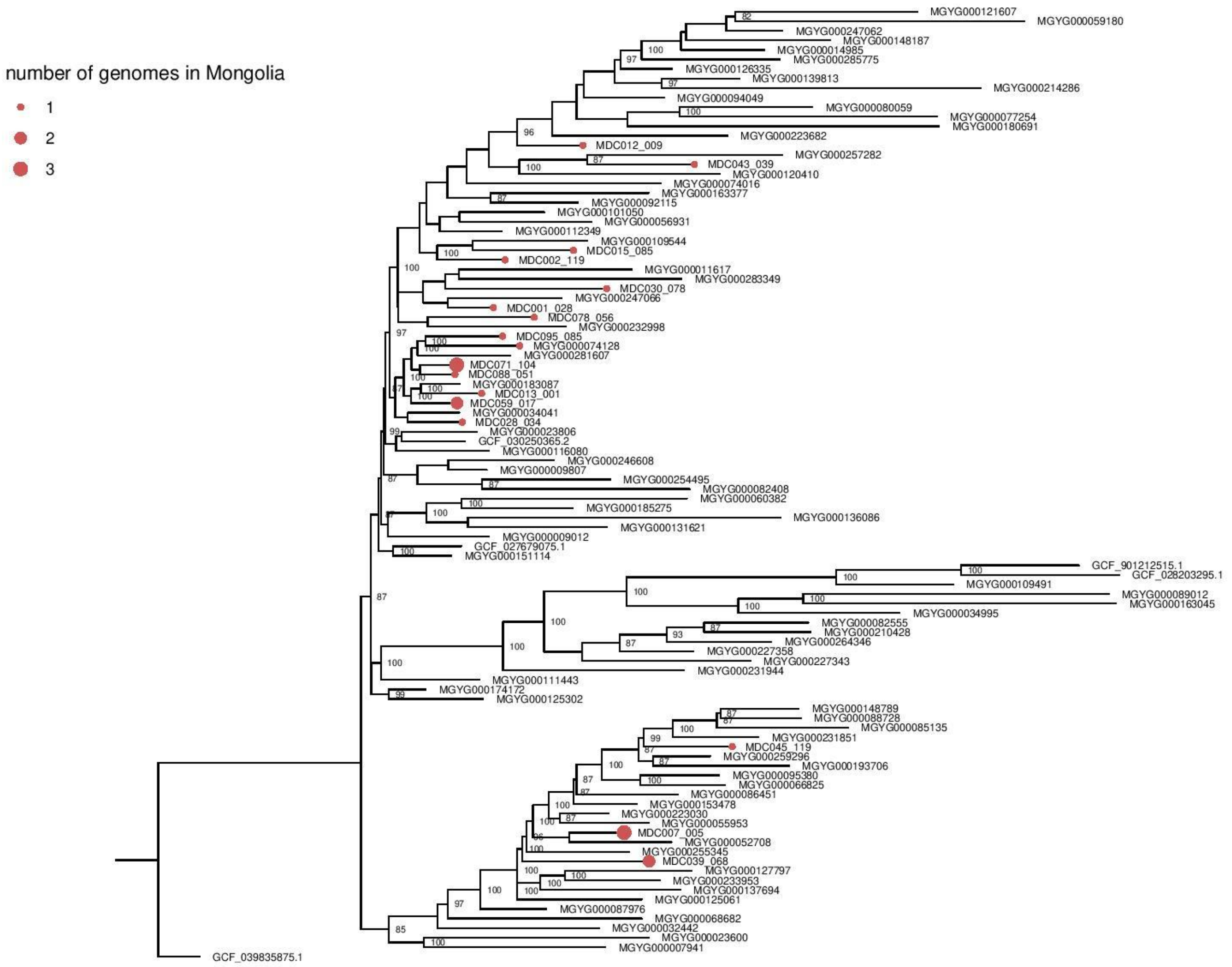

Figure S4. Maximum-likelihood phylogenetic tree of *B. catenulatum*. The tree was constructed using 1,996 core genes from 314 publicly available genomes together with 23 genomes identified in Mongolian participants. Red circles indicate Mongolian genomes, and circle size corresponds to the number of genomes detected. Numbers at the nodes represent bootstrap support values (%). The phylogenetic analysis included 1,055 core genes and 941 outgroup genes.

Bifidobacterium longum: Phylogenetic tree including 65 Mongolian genomes

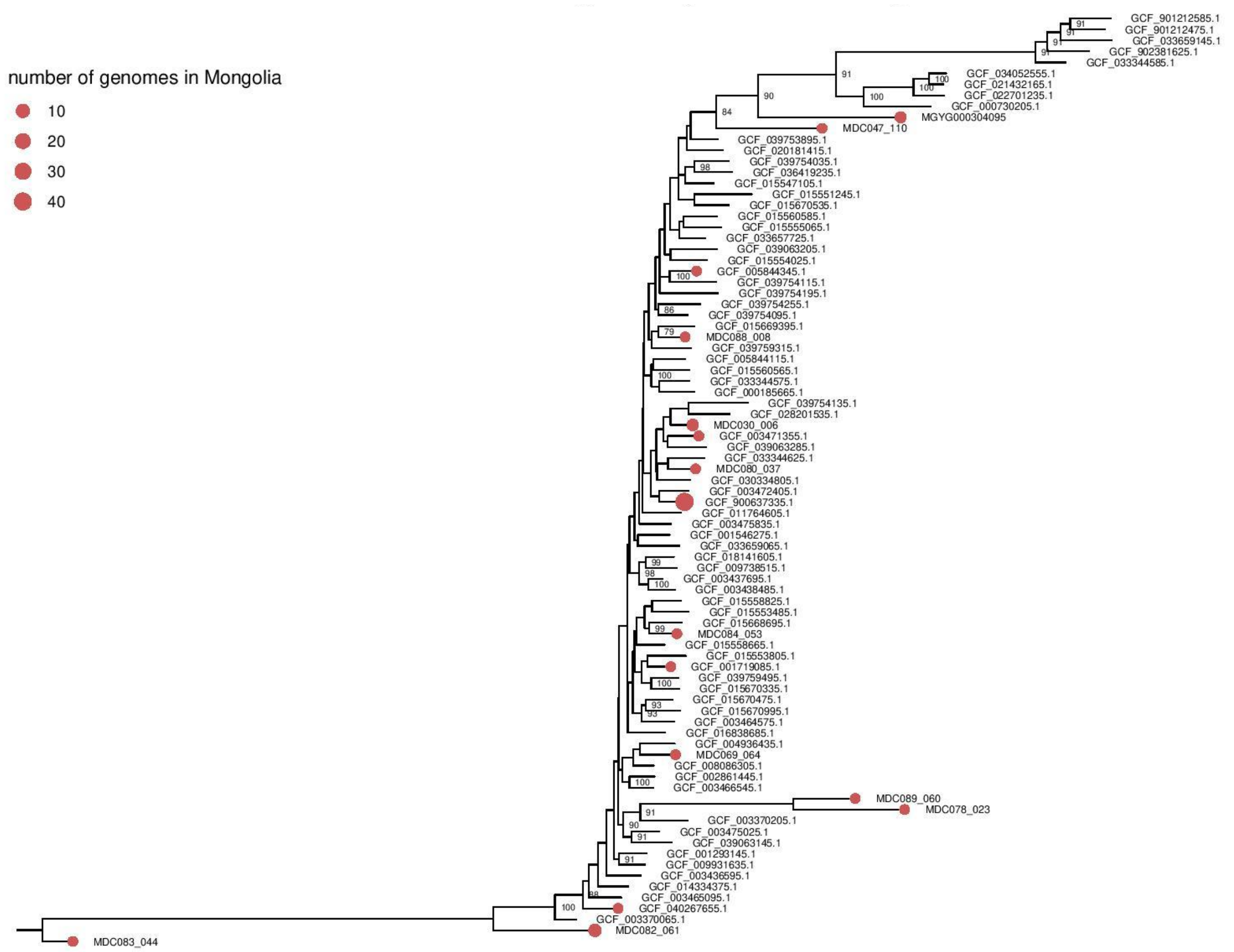

Figure S5. Maximum-likelihood phylogenetic tree of *B. longum*. The tree was constructed using 1,006 core genes from 203 publicly available genomes together with 65 genomes identified in Mongolian participants. Red circles indicate Mongolian genomes, and circle size corresponds to the number of genomes detected. Numbers at the nodes represent bootstrap support values (%). The phylogenetic analysis included 924 core genes and 82 outgroup genes.

***Bifidobacterium pseudocatenulatum*: Phylogenetic tree including 20 Mongolian genomes**

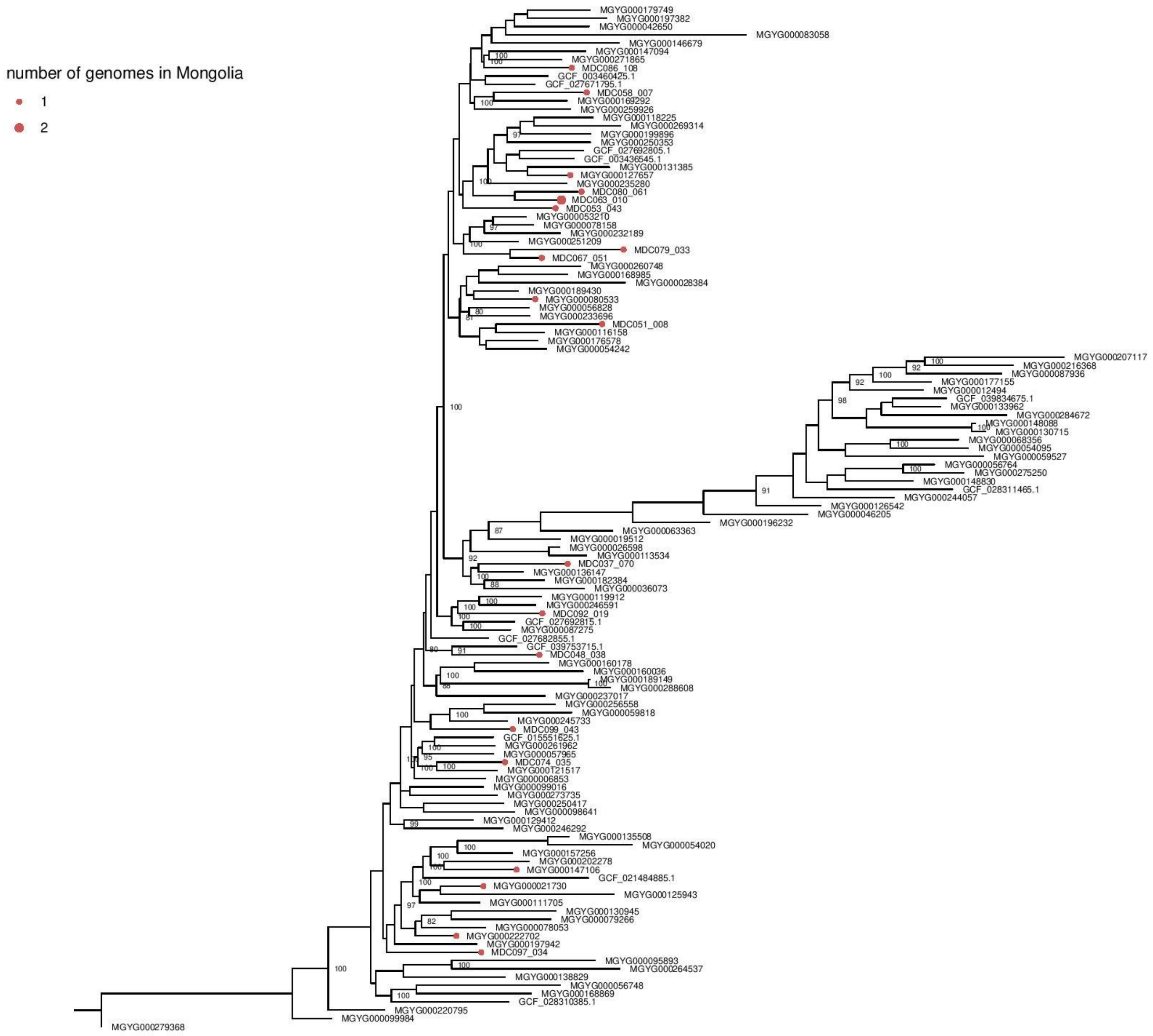

Figure S6. Maximum-likelihood phylogenetic tree of *B. pseudocatenulatum*. The tree was constructed using 1,665 core genes from 1,324 publicly available genomes together with 20 genomes identified in Mongolian participants. Red circles indicate Mongolian genomes, and circle size corresponds to the number of genomes detected. Numbers at the nodes represent bootstrap support values (%). The phylogenetic analysis included 908 core genes and 757 outgroup genes.

Table S1. Spearman correlation analysis between dairy intake variables and the relative abundance of *Bifidobacterium* species. *p* values were adjusted using the Benjamini–Hochberg false discovery rate (FDR) method.

№	Dairy	Species	Rho	*p*	FDR
1	Factory_Milk	*B.longum_RA*	0.038	0.705	0.772
2	Factory_Yogurt	*B.longum_RA*	-0.069	0.493	0.678
3	Fermented_Horse_Milk	*B.longum_RA*	-0.229	0.022	0.101
4	Fermented_Camel_Milk	*B.longum_RA*	-0.086	0.397	0.616
5	Homemade_Yogurt	*B.longum_RA*	0.036	0.720	0.772
6	Traditional_dairy_g	*B.longum_RA*	-0.113	0.263	0.434
7	Fermented_dairy_g	*B.longum_RA*	-0.063	0.532	0.678
8	Nonfermented_dairy_g	*B.longum_RA*	-0.025	0.803	0.817
9	Factory_Milk	*B.adolescentis_RA*	0.035	0.731	0.772
10	Factory_Yogurt	*B.adolescentis_RA*	0.061	0.544	0.678
11	Fermented_Horse_Milk	*B.adolescentis_RA*	-0.235	0.019	0.094
12	Fermented_Camel_Milk	*B.adolescentis_RA*	0.123	0.223	0.391
13	Homemade_Yogurt	*B.adolescentis_RA*	0.196	0.050	0.166
14	Traditional_dairy_g	*B.adolescentis_RA*	-0.088	0.385	0.616
15	Fermented_dairy_g	*B.adolescentis_RA*	0.044	0.664	0.765
16	Nonfermented_dairy_g	*B.adolescentis_RA*	-0.160	0.112	0.299
17	Factory_Milk	*B.bifidum_RA*	-0.027	0.793	0.817
18	Factory_Yogurt	*B.bifidum_RA*	0.043	0.669	0.765
19	Fermented_Horse_Milk	*B.bifidum_RA*	-0.125	0.214	0.391
20	Fermented_Camel_Milk	*B.bifidum_RA*	0.155	0.123	0.314
21	Homemade_Yogurt	*B.bifidum_RA*	0.161	0.111	0.299
22	Traditional_dairy_g	*B.bifidum_RA*	-0.014	0.888	0.888
23	Fermented_dairy_g	*B.bifidum_RA*	0.037	0.712	0.772
24	Nonfermented_dairy_g	*B.bifidum_RA*	-0.117	0.247	0.419
25	Factory_Milk	*B.angulatum_RA*	-0.289	0.003	0.028
26	Factory_Yogurt	*B.angulatum_RA*	-0.323	0.001	0.014
27	Fermented_Horse_Milk	*B.angulatum_RA*	-0.133	0.186	0.372
28	Fermented_Camel_Milk	*B.angulatum_RA*	0.177	0.078	0.242
29	Homemade_Yogurt	*B.angulatum_RA*	0.293	0.003	0.028
30	Traditional_dairy_g	*B.angulatum_RA*	0.270	0.007	0.046
31	Fermented_dairy_g	*B.angulatum_RA*	0.241	0.016	0.094
32	Nonfermented_dairy_g	*B.angulatum_RA*	0.056	0.578	0.703
33	Factory_Milk	*B.catenulatum_RA*	-0.143	0.157	0.352
34	Factory_Yogurt	*B.catenulatum_RA*	-0.148	0.142	0.331
35	Fermented_Horse_Milk	*B.catenulatum_RA*	-0.137	0.174	0.361
36	Fermented_Camel_Milk	*B.catenulatum_RA*	0.069	0.494	0.678
37	Homemade_Yogurt	*B.catenulatum_RA*	0.331	0.001	0.014
38	Traditional_dairy_g	*B.catenulatum_RA*	0.344	0.000	0.013
39	Fermented_dairy_g	*B.catenulatum_RA*	0.302	0.002	0.025
40	Nonfermented_dairy_g	*B.catenulatum_RA*	0.073	0.471	0.678
41	Factory_Milk	*B.dentium_RA*	-0.221	0.027	0.117
42	Factory_Yogurt	*B.dentium_RA*	-0.204	0.041	0.154
43	Fermented_Horse_Milk	*B.dentium_RA*	-0.048	0.632	0.753
44	Fermented_Camel_Milk	*B.dentium_RA*	-0.066	0.517	0.678
45	Homemade_Yogurt	*B.dentium_RA*	0.128	0.204	0.391
46	Traditional_dairy_g	*B.dentium_RA*	0.237	0.017	0.094
47	Fermented_dairy_g	*B.dentium_RA*	0.199	0.047	0.166
48	Nonfermented_dairy_g	*B.dentium_RA*	0.123	0.222	0.391
49	Factory_Milk	*B.pseudocatenulatum_RA*	0.346	0.000	0.013
50	Factory_Yogurt	*B.pseudocatenulatum_RA*	0.137	0.174	0.361
51	Fermented_Horse_Milk	*B.pseudocatenulatum_RA*	-0.062	0.542	0.678
52	Fermented_Camel_Milk	*B.pseudocatenulatum_RA*	-0.084	0.407	0.616
53	Homemade_Yogurt	*B.pseudocatenulatum_RA*	-0.167	0.097	0.285
54	Traditional_dairy_g	*B.pseudocatenulatum_RA*	-0.218	0.029	0.117
55	Fermented_dairy_g	*B.pseudocatenulatum_RA*	-0.151	0.134	0.326
56	Nonfermented_dairy_g	*B.pseudocatenulatum_RA*	-0.063	0.535	0.678
